# Divergent Neural Activity in the VLPO During Anesthesia and Sleep

**DOI:** 10.1002/advs.202203395

**Published:** 2022-12-03

**Authors:** Mengqiang Luo, Xiang Fei, Xiaotong Liu, Zikang Jin, Yingwei Wang, Min Xu

**Affiliations:** ^1^ Department of Anesthesiology Huashan Hospital Fudan University Shanghai 200040 China; ^2^ Institute of Neuroscience State Key Laboratory of Neuroscience Center for Excellence in Brain Science and Intelligence Technology Chinese Academy of Sciences Shanghai 200031 China; ^3^ Shanghai Center for Brain Science and Brain‐Inspired Intelligence Technology Shanghai 201210 China

**Keywords:** general anesthesia, micro‐endoscopic imaging, sleep, ventrolateral preoptic nucleus

## Abstract

The invention of general anesthesia (GA) represents a significant advance in modern clinical practices. However, the exact mechanisms of GA are not entirely understood. Because of the multitude of similarities between GA and sleep, one intriguing hypothesis is that anesthesia may engage the sleep‐wake regulation circuits. Here, using fiber photometry and micro‐endoscopic imaging of Ca^2+^ signals at both population and single‐cell levels, it investigates how various anesthetics modulate the neural activity in the ventrolateral preoptic nucleus (vLPO), a brain region essential for the initiation of sleep. It is found that different anesthetics primarily induced suppression of neural activity and tended to recruit a similar group of vLPO neurons; however, each anesthetic caused comparable modulations of both wake‐active and sleep‐active neurons. These results demonstrate that anesthesia creates a different state of neural activity in the vLPO than during natural sleep, suggesting that anesthesia may not engage the same vLPO circuits for sleep generation.

## Graphical abstract

It has long been thought that general anesthesia (GA) may use natural sleep regulation circuits because of similarities in the body and brain during GA and sleep. However, it is found that in the core sleep circuit, the neural activity induced by anesthetics is very different compared to natural sleep, suggesting that they are distinct brain states.

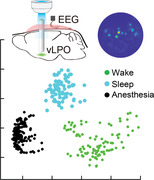

## 1 Introduction

General anesthesia (GA) represents a drug‐induced reversible state of unconsciousness, playing a critical role in modern medicine since its invention in 1846.^[^ ^1^, ^2^, ^3^, ^4^ ^]^ However, the exact mechanisms of GA are not entirely clear.^[^ ^5^, ^6^, ^7^ ^]^ Previous studies have revealed multiple receptors as targets for various general anesthetics, including the *γ*‐aminobutyric acid (GABA) receptors and the N‐methyl‐D‐aspartic acid (NMDA) receptors.^[^ ^8^, ^9^, ^10^, ^11^, ^12^ ^]^ Meanwhile, at the neural circuitry level, it is less clear which neural pathway mediates the effects of GA.^[^ ^1^, ^11^, ^13^ ^]^

Because of the similarity in the physiology signs and EEG patterns during GA and sleep (particularly the slow‐wave sleep, or non‐rapid eye movement sleep, NREM),^[^ ^3^ ^]^ it has long been believed that GA may engage the natural sleep‐wake regulation circuits.^[^ ^1^, ^14^, ^15^, ^16^ ^]^ Studies using immediate‐early genes (IEGs)‐related techniques show that many brain regions (e.g., the ventrolateral preoptic nucleus, vLPO; the lateral habenula; the supraoptic nucleus) that are involved in the sleep‐wake regulation are activated during GA;^[^ ^15^, ^16^, ^17^, ^18^, ^19^, ^20^, ^21^ ^]^ neuronal activity within these brain regions can be directly modulated by the application of some anesthetics in ex‐vivo brain slice experiments.^[^ ^16^, ^19^, ^22^, ^23^ ^]^ Furthermore, manipulating the neural activity of multiple sleep‐wake circuits was reported to affect the induction or emergence of GA—e.g., vLPO,^[^ ^19^, ^24^ ^]^ the tuberomammillary nucleus,^[^ ^25^ ^]^ basal forebrain,^[^ ^26^, ^27^, ^28^, ^29^ ^]^ nucleus accumbens,^[^ ^30^, ^31^ ^]^ ventral tegmental area,^[^ ^32^, ^33^, ^34^, ^35^ ^]^ locus coeruleus,^[^ ^29^, ^36^ ^]^ parabrachial nucleus,^[^ ^37^, ^38^, ^39^ ^]^ and thalamus.^[^ ^40^ ^]^ However, these experiments often do not provide critical data directly comparing neural activity during anesthesia and sleep; it is thus still unclear whether and how anesthesia engages the same neural population responsible for the generation of sleep. In addition, many anesthetics, such as isoflurane and propofol, primarily enhance inhibitory transmission and reduce neural activity,^[^ ^12^ ^]^ while another anesthetic, ketamine, works by producing network dissociation.^[^ ^41^ ^]^ It is unclear whether these general anesthetics affect sleep‐promoting circuits similarly.

In the current study, we tested the long‐standing hypothesis that anesthesia recruits sleep‐promoting neural circuits by directly comparing the activity of the GABAergic neurons in the vLPO (vLPO^GABA^) during anesthesia and natural sleep. The vLPO^GABA^ neurons were selected because of their well‐documented role in sleep control.^[^ ^16^, ^42^, ^43^, ^44^, ^45^, ^46^, ^47^, ^48^ ^]^ We recorded the neural activity of vLPO^GABA^ neurons at the population level using fiber photometry and at the single‐neuron level using micro‐endoscopic imaging when mice were treated with four commonly‐used anesthetics as well as during the natural sleep‐wake cycle. A comparison of the activity modulation in the same group of recorded neurons revealed divergent states of the vLPO circuits during anesthesia and sleep.

## 2 Results

### 2.1 Suppression of Population Neural Activity in the vLPO by Anesthetics

**Figure 1:**
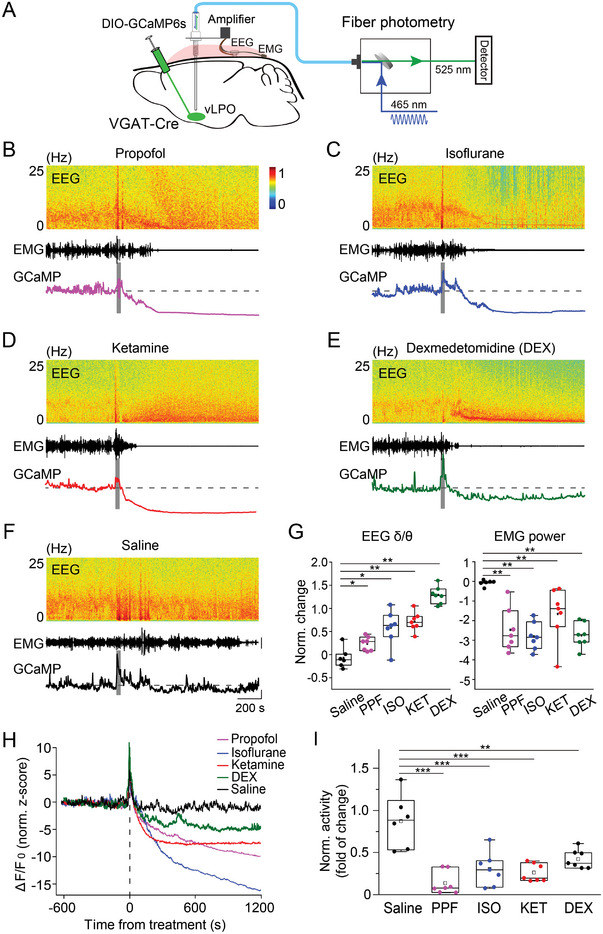
Anesthetics suppress the population Ca^2+^ signal of GABAergic neurons in the vLPO. A) Schematic diagram depicting fiber photometry recording of population Ca^2+^ signal of GABAergic neurons in the vLPO. Note that the virus was injected with no angle. B–F) Representative recordings showing the change in the population Ca^2+^ signal and EEG/EMG after the application of each anesthetic or control, as indicated in each panel. (Top to bottom) EEG power spectrogram, EMG (scale, 0.5 mV), and GCaMP signal (Scale, 5 normalized z‐score, and 200 s). The gray bar indicates the timing of each treatment. The Dash line indicates the baseline. G) Statistical summary of changes in EEG *δ*/*θ* (left) and EMG after each treatment. The box plot shows a ≈25% to 75% range, the line shows a range within the 1.5 interquartile range (IQR), and the dot represents the median.*n* = 7, 7, 7, 7, and 6 sessions from six mice for PPF, ISO, KET, DEX, and saline, respectively. EEG: *p* = 0.027, 0.012, 0.003, and 0.003, EMG: *p* = 0.003, 0.003, 0.003, and 0.003 for PPF, ISO, KET, and DEX, respectively (comparing with the saline condition, Wilcoxon rank‐sum test). In this and all subsequent Figs, summary data are expressed as the mean ± s.e.m. H) Averaged Ca^2+^ signal from each experiment (*n* = 7, 7, 7, 7, and 6 sessions from 6 mice for PPF, ISO, KET, DEX, and saline, respectively). I) Statistical summary for change of Ca^2+^ signal after each treatment. Each dot represents one experiment. The box plot shows a ≈25% to 75% range, the line shows a range within 1.5 IQR, and the dot represents the median. *n* = 7, 7, 7, 7, and 6 sessions from six mice for PPF, ISO, KET, DEX, and saline, respectively. ***p < 0.0001 for PPF, ISO, and KET, respectively; **p = 0.0014 for DEX (comparing with the saline condition, one‐way ANOVA with post hoc Tukey's test).

To examine how anesthetics modulate the neural activity of the sleep circuits, we first measured the changes in population Ca^2+^ activity from the GABAergic neurons in the vLPO. We injected an adeno‐associated virus (AAV) expressing the Cre‐dependent Ca^2+^ indicator GCaMP6s^[^ ^49^ ^]^ into the vLPO of the VGAT‐IRES‐Cre mice,^[^ ^50^ ^]^ and measured the fluorescence signal using fiber photometry two weeks after injection (**Figure** **1**A and Figure S1A,B, Supporting Information).^[^ ^51^ ^]^ The use of fiber photometry allows for longitudinal recording of Ca^2+^ activity of the same group of neurons in multiple experimental conditions spanning several days.

We found that the population Ca^2+^ activity of the vLPO^GABA^ neurons was strongly suppressed shortly after the application of each anesthetic or sedative (Figure 1B–F)—including propofol (PPF), isoflurane (ISO), ketamine (KET), and dexmedetomidine (DEX) (for simplicity, hereinafter, we use “anesthetics” to refer to all anesthetics and sedatives used in this study)—although the underlying acting mechanisms of these anesthetics are different.^[^ ^1^, ^5^, ^6^, ^11^ ^]^ In our experiments, we used the does for different anesthetics that were commonly used in mouse experiments to induce loss‐of‐consciousness (PPF, 180 mg kg^−1^, i.p.; KET, 100 mg kg^−1^, i.p.; DEX, 100–150 µg kg^−1^, i.p.; ISO, induction, 2.5%, maintenance, 1%).^[^ ^16^, ^21^, ^32^ ^]^

To quantitatively describe the brain state induced by these anesthetics, we also recorded the EMG (from neck muscles) and EEG (occipital cortex) of the mice. Application of the four anesthetics all caused significant changes in the patterns of both EMG and EEG, with substantial suppression of EMG power (*p* < 0.004 for all anesthetics, Wilcoxon rank‐sum test) and an increase in the ratio between EEG delta and theta power (*δ*/*θ*) (*p* < 0.03 for all anesthetics, Wilcoxon rank‐sum test) (Figure 1G), consistent with previous reports.^[^ ^6^ ^]^ In the state space defined by EMG power and EEG *δ*/*θ* ratio, the brain state before and after applying each anesthetic formed two distinct clusters (Figure S2A and Movie S1, Supporting Information). To quantify the difference between the brain state during wakefulness and that evoked by the four anesthetics, the Davies–Bouldin index (DBI) was used to evaluate the separation of the two clusters before and after anesthesia induction (Figure S2B, Supporting Information). Compared with the commonly used behavior indicators for anesthesia, such as the loss of righting reflex (LORR), the DBI can provide a quantitative measurement of brain states after anesthesia, with a high temporal resolution and minimal physical stimulation to the animal. The use of DBI also reduced variation due to differences in mouse sensitivity to the four anesthetics and ensured that the data used in our analyses were from a relatively homogeneous state of anesthesia.

We then used the DBI to determine the period of recordings after applications of each anesthetic to analyze the modulation of neural activity, such that the resulting DBI was much smaller than that of the control conditions (saline injection) (Figure S3A, Supporting Information). The DBI after application of each anesthetic was typically very small—DBI (mean ± s.e.m.): 0.21 ± 0.05, 0.16 ± 0.02, 0.29 ± 0.06, and 0.15 ± 0.02 for PPF, ISO, KET, and DEX, respectively; DBI for control condition: 0.94 ± 0.09. These selected periods showed minimal EMG activity (Figure S3B, Supporting Information), indicating a lack of movement during anesthesia. This lack of movement was also confirmed using the behavioral camera capturing the facial movements of mice (Figure S3C, Supporting Information), which was recently shown as a measure of the loss of consciousness in head‐fixed mice.^[^ ^52^ ^]^

After we used the DBI to determine the period of recordings with adequate depth of anesthesia (see Experimental Section), we compared the modulation of the population Ca^2+^ activity caused by each anesthetic and found that all four anesthetics caused a significantly smaller Ca^2+^ signal than that in the control conditions (Figure 1H,I, *p* < 0.0015 for all four anesthetics, one‐way ANOVA with post hoc Tukey's test). These results suggested that anesthesia is primarily associated with inhibiting the population Ca^2+^ activity of the vLPO GABAergic neurons.^[^ ^28^, ^30^, ^38^, ^53^ ^]^

### 2.2 Diverse Modulation of Single‐Neuron Activity in the vLPO by Anesthetics

**Figure 2:**
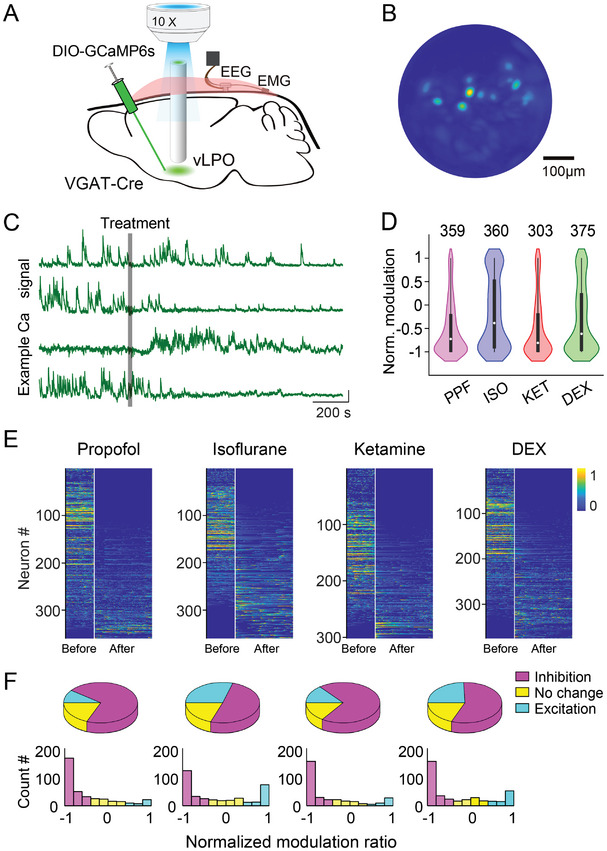
Anesthetics induce diverse modulation of Ca^2+^ signals from individual GABAergic neurons in the vLPO. A) Schematic diagram depicting micro‐endoscopic recording of population Ca^2+^ signal of GABAergic neurons in the vLPO. The GCaMP6‐expressing neurons were imaged using an epifluorescence microscope via an implanted GRIN lens. B) Representative field of view from an example imaging session. C) Example Ca^2+^ signal (raw trace) from four simultaneously recorded neurons responding to the application of anesthetics. Each line is one neuron. The gray bar indicates the timing of the treatment. Scale bar, 10% (*ΔF*/*F*_0_) and 200 s. D) Statistical summary of the normalized modulation by each anesthetic. The normalized modulation index (NMI) was defined as the Ca^2+^ signal (area under curve, AUC) after each treatment (10 min after each treatment, determined by DBI) minus the Ca^2+^ signal during the baseline period (5 min before each treatment) divide by the sum of the two signals. Such that neurons inhibited by anesthetics will have a negative NMI, and the range of the NMI will be ≈ −1 to 1. The violin plots were a combination of kernel density plot (KDE) and box plot. The box plot shows a 25%∼75% range, the line shows a range within 1.5 IQR, and the dot represents the median. *n* = 359, 360, 303, and 375; *p* < 0.0001 for all comparison (Wilcoxon signed‐rank test). E) Heat map showing the Ca^2+^ signal of each neuron during baseline and the period with an adequate level of anesthesia (determined using the DBI measurement). The recording periods were spliced together, and the white line indicates the splicing point (same for all heatmap plots in the following figures). Neurons were sorted by the NMI. F) Pie chart showing the percentage of neurons modulated by each anesthetic (up). Distribution of the NMI induced by each treatment (low).

We next examined the modulation of the vLPO neural activity at a single‐cell level, because previous research showed that vLPO neurons show diverse activity modulation during the sleep‐wake cycle, with the sleep‐active neurons spatially intermingling with wake‐active neurons.^[^ ^46^, ^54^ ^]^ We recorded the Ca^2+^ activity of individual vLPO^GABA^ neurons using a micro‐endoscopic imaging technique,^[^ ^55^, ^56^, ^57^ ^]^ in which GCaMP6s‐expressing neurons were imaged using an epifluorescence microscope via an implanted gradient refractive index (GRIN) lens (**Figure** **2**A,B, Figure S4A, B, and Movie S2, Supporting Information). The micro‐endoscopic imaging method can provide stable access to the activity of the same group of neurons with a single‐cell resolution for multiple weeks, which is critical for our longitudinal comparisons of activity modulation by the four anesthetics.

The CNMF‐E algorithm^[^ ^58^ ^]^ was used to extract the Ca^2+^ signal of each neuron from the raw imaging data. In our micro‐endoscopic imaging, we used the DBI as a metric to identify the recording period with adequate depth of anesthesia to analyze the modulation of neural activity by each anesthetic. The four anesthetics produced comparable modulation to the brain state as measured by DBI (0.49 ± 0.03, 0.33 ± 0.05, 0.42 ± 0.06, and 0.42 ± 0.05 for PPF, ISO, KET, and DEX, respectively; mean ± s.e.m.; *p* = 0.17, one‐way ANOVA). We then examined the modulation of neural activity by calculating the normalized modulation index (NMI), which was defined as the ratio between the difference of Ca^2+^ signal (measured by the area under the curve, AUC) after and before the application of each anesthetic and the sum of the Ca^2+^ signal—a negative NMI thus represents neural activity inhibited by the anesthetics.

We performed Ca^2+^ imaging from 10 mice while applying the four anesthetics and totally extracted 1397 neurons (303–375 in each condition). At the single‐neuron level, we observed diverse modulation of the Ca^2+^ signal in vLPO^GABA^ neurons after applying each anesthetic (Figure 2C), in contrast to the uniform inhibition observed in our previous recording of the population Ca^2+^ signal using fiber photometry. However, consistent with recordings at the population level, the averaged activity of individual vLPO^GABA^ neurons was also significantly suppressed by the application of the four anesthetics as quantified using the NMI (Figure 2D)—NMI (median ± s.e.m.): PPF, −0.73 ± 0.03; ISO, −0.39 ± 0.04; KET, −0.81 ± 0.04; DEX, −0.62 ± 0.04; *p* < 0.0001 for all groups, Wilcoxon sign‐rank test for all four conditions). We also observed large variations in the NMI evoked by all four anesthetics (Figure 2D; the standard deviation of each group: PPF, 0.58; ISO, 0.76; KET, 0.64; DEX, 0.72), consistent with the diverse modulations of the Ca^2+^ signal observed during the recordings.

When we examined the modulation of individual vLPO^GABA^ neurons, we used the hierarchical clustering method to classify the response patterns of these neurons to the applications of the four anesthetics. The vLPO^GABA^ neurons formed three groups in the cluster dendrogram—Neurons that were inhibited, excited, or insensitive to the application of these anesthetics (Figure S5, Supporting Information). The majority of recorded neurons were inhibited (NMI < ‐0.33) (Figure 2E,f; the percentage of inhibited neurons: PPF, 71%; ISO, 51%; KET, 70%; DEX, 58%). However, we also observed that a significant portion of the neurons was excited (NMI > 0.33) (Figure 2E,F; the percentage of excited neurons: PPF, 10%; ISO, 29%; KET, 14%; DEX, 23%). Interestingly, in the distribution of the NMI, many neurons showed extreme modulations (with NMI close to 1 or −1) in both inhibited and excited groups (Figure 2F), suggesting a high sensitivity of these neurons in responding to the application or removal of each anesthetic. These results revealed a diverse modulation of individual vLPO^GABA^ neurons underneath the uniformed inhibition observed in the population recording.

### 2.3 Different Anesthetics Engage Similar yet Different vLPO Neural Populations

Given the diverse modulation evoked by the four anesthetics, it is interesting to find out whether different anesthetics cause similar modulation to the same vLPO^GABA^ neuron. To address this question, we manually aligned the imaging data in each condition and only used neurons recorded in multiple experiment conditions for further analysis (Figure S6, Supporting Information). Of all the 1397 neurons, we identified 172–197 neurons from 10 mice that were imaged under more than two anesthesia conditions.

**Figure 3:**
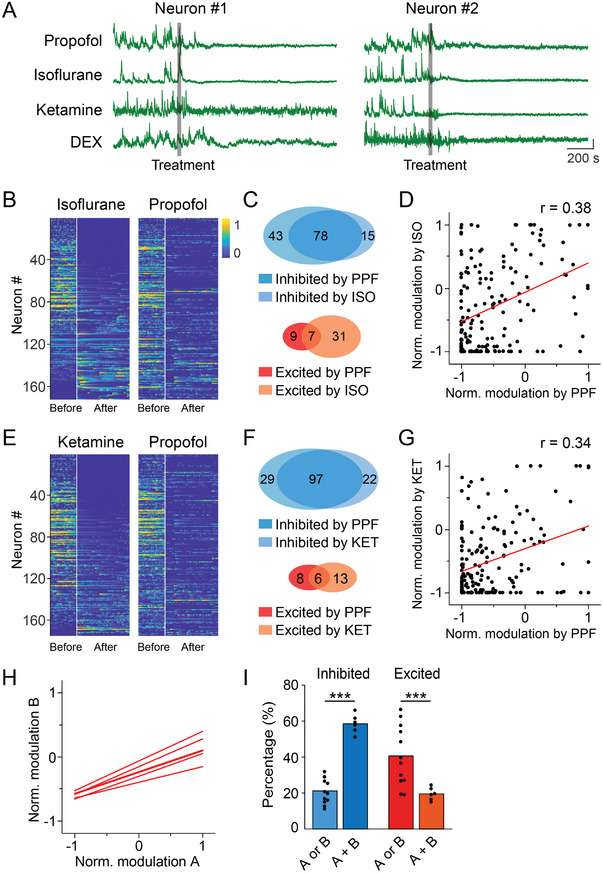
Different anesthetics engage similar vLPO GABAergic neurons. A) Example Ca^2+^ signal from the same neuron responding to the application of four anesthetics. Each line represents one condition. The gray bar indicates the timing of the treatment. Scale bar, 10% (*ΔF*/*F*_0_) and 200 s. B) Heat map showing the Ca^2+^ signal of each neuron during baseline and the period with an adequate level of anesthesia modulation by ISO and PPF. Neurons were sorted by the NMI in ISO condition. C) Venn diagram of the number of neurons inhibited by PPF or ISO (up) or neurons excited by PPF or ISO (low). D) Scatter plot showing the correlation between the modulation evoked by PPF and ISO (*n* = 172). Each dot is one neuron. The red line is the linear fit of the data. E–G) Same as B‐D, respectively, except the comparison was between PPF and KET (*n* = 175). Neurons in the heat map were sorted by the NMI in KET condition. H) A summary showing the linear fit of all pairwise comparisons. I) Statistical summary for the percentage of neurons showing the same or different modulation by various anesthetics. Both inhibited 58.0 ± 2.1%, only inhibited by one anesthetic 21.0 ± 2.0%; both excited 19.4 ± 1.2%, only excited by one anesthetic 40.3 ± 4.8%, mean ± s.e.m., *p* < 0.0001 and *p* = 0.0085 for the inhibited and excited group, respectively (Student's *t*‐test).

We first compared the modulation of the same neuron by PPF and ISO, because they both induce anesthesia through a similar mechanism by enhancing GABAergic transmission.^[^ ^5^, ^6^, ^12^ ^]^ We indeed observed neurons showing similar responses to the application of both anesthetics (**Figure** **3**A). In all the 172 neurons imaged in the two conditions, the majority showed a similar modulation—78 out of 121 neurons inhibited by PPF were also inhibited by ISO; 78 out of 93 neurons inhibited by ISO were also inhibited by PPF (Figure 3B,C). The same trend of overlap was also observed in neurons excited by both anesthetics (Figure 3B,C). However, the modulation of the same neuron by the two anesthetics was quantitatively different; thus, the correlation coefficient between the modulations evoked by ISO and PPF was moderate (Pearson's *r* = 0.38, *p* < 0.001) (Figure 3D). To be noted, this moderate correlation between different treatment conditions was unlikely caused by experimental variations because we observed a strong correlation in the modulation between different repeats of the same treatment on different days (Figure S7A,B, Supporting Information).

We next investigated whether anesthetics with different working mechanisms (e.g., targeting GABA receptors or NMDA receptors) also induced similar modulation of the same neuron by examining the modulation caused by PPF and KET.^[^ ^5^, ^6^, ^12^ ^]^ PPF and KET also caused similar yet quantitatively different modulations as that observed in the comparison between the PPF and ISO conditions—58.9% (103 out of 175) neurons showed the same modulations, and the correlation coefficient equaled 0.34 (*p* < 0.001) (Figure 3E–G). In fact, the same trend was also observed in all pairwise comparisons of the modulations caused by the four anesthetics in our experiments (Figure 3H,I and Figure S8, Supporting Information) (51.8 ± 1.9% neurons had the same modulation, Pearson's *r* = 0.34 ± 0.01, mean ± s.e.m., *p* < 0.001 for all comparisons). Thus, although the four anesthetics caused quantitatively different modulations to the same vLPO^GABA^ neuron, they appeared to recruit a similar group of neurons, despite the difference in each anesthetic's mechanism of action.

### 2.4 Anesthesia and Sleep Evoke Divergent Modulation to the vLPO^GABA^ Neural Population

We have shown that various anesthetics seem to engage a similar group of neurons, so we wonder whether these neurons have correlated activity in the sleep‐wake cycle. Specifically, are the neurons inhibited or excited by each anesthetic corresponding to the wake‐active or sleep‐active vLPO neurons, respectively?

**Figure 4:**
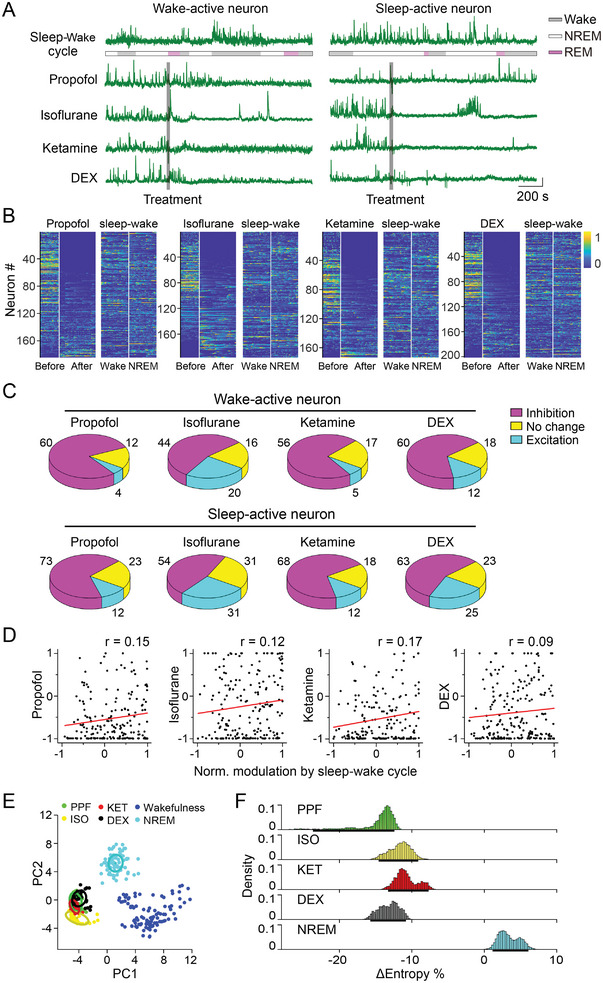
Anesthesia and sleep cause distinct modulation of the vLPO GABAergic neurons. A) Example Ca^2+^ signal from the same neuron during the application of four anesthetics and that during the natural sleep‐wake cycle. Each line represents one condition. The gray bar indicates the timing of the treatment. Color code represents the brain states during the sleep‐wake cycle (gray, wakefulness; yellow, NREM; blue, REM), Scale bar, 10% (*ΔF*/*F*_0_) and 200 s. B) Heat map showing modulation by each anesthetic and during the sleep‐wake cycle. Neurons were sorted by the NMI of applying each anesthetic. (*n* = 184, 196, 176, and 201 for PPF, ISO, KET, and DEX, respectively). C) Pie chart showing the modulation of the wake‐active neurons by each anesthetic (up) and modulation of the sleep‐active neurons (low). D) Scatter plot showing the correlation between the modulation evoked by each anesthetic and the sleep‐wake cycle (*n* = 184, 196, 176, and 201 for PPF, ISO, KET, and DEX, respectively). Each dot is one neuron. The red line is the linear fit of the data. E) Visualization of the neural activity in the state space using PCA. Neural activity under various conditions was color‐coded. The plot was constructed using data from 123 neurons that were recorded in all five conditions; each dot represents population neural activity in a 15‐s bin. F) Change in entropy (comparing with entropy during wakefulness) during NREM sleep and anesthesia induced by four anesthetics. We used the dataset in(B) to calculate the population entropy of the vLPO GABAergic neurons (see Experimental Section). Δ_entropy_: −14.6%, 95% CIs [−23.6% ∼ −12.1%]; −11.7%, [≈ −14.6% to −9.1%]); ‐10.8%, [≈ −13.3% to −7.7%]); −13.1%, [≈ −15.7% to −10.8%]); and 3.4%, [≈1.1% to 6.1%] for PPF, ISO, KET, DEX, and NREM conditions, respectively.

To answer this question, we examined how the four anesthetics modulate wake‐active and sleep‐active vLPO^GABA^ neurons. Ca^2+^ signals from the same neuron were recorded during multiple days when the mouse was subjected to anesthesia and during the natural sleep‐wake cycle. We identified 757 neurons (176–201 neurons in each condition) from 10 mice that were imaged under both anesthesia and sleep. There was diverse modulation in both wake‐active neurons and sleep‐active neurons by all four anesthetics used in our study (**Figure** **4**A–C). In the wake‐active vLPO^GABA^ neurons (defined as neurons that have positive NMI during the sleep‐wake cycle; neurons that have negative NMI were defined as sleep‐active neurons), a significant portion (on average, 67.8%) was inhibited by the application of various anesthetics, and PPF induced the most prominent inhibition (78.9%), while ISO caused the smallest inhibition (55.0%) (Figure 4A–C). In the sleep‐active neurons, the same trend was also observed—on average, 59.6% was inhibited, and KET induced the most prominent inhibition (69.4%), while ISO caused the smallest inhibition (46.6%) (Figure 4C).

We next compared the correlation between the modulations evoked by the four anesthetics and that by the sleep‐wake cycle for individual neurons and found no significant correlations (Pearson's *r* = 0.15, 0.12, 0.17, and 0.09 for PPF, ISO, KET, and DEX condition, respectively) (Figure 4D). These results showed that the four anesthetics caused comparable modulation (primarily inhibition) to both sleep‐active neurons and wake‐active neurons, suggesting that activity patterns of the vLPO^GABA^ neurons during anesthesia were different from that during natural sleep.

To further illustrate the divergent states of neural activity in the vLPO during anesthesia and sleep, we used principal component analysis (PCA) to capture the major variances of the neural activity under various conditions and constructed a 2D state space of the vLPO^GABA^ neurons (Figure 4E). We identified 123 neurons from 10 mice that were recorded under all four anesthesia conditions and during the sleep‐wake cycle to perform the PCA. Thus, at each given time, the state of the vLPO^GABA^ neurons was described by a linear vector consisting of the activity of all 123 neurons. After dimension reduction using PCA, we visualized the population activity in a 2D space defined by the first two principal components (Figure 4E). We found that the vLPO^GABA^ activity during wakefulness, sleep, and anesthesia formed three distinct clusters (Figure 4E). To be noted, the PCA results were not affected by the length of the time bin used to calculate the activity of each neuron, because a different time bin of 30 sec resulted in a similar conclusion (Figure S9, Supporting Information). This result is consistent with our above result showing that different anesthetics caused similar modulation to the vLPO^GABA^ neurons, but anesthesia and sleep induced divergent modulations, further supporting that the vLPO neural activity was in different states during anesthesia and natural sleep.

Finally, we evaluated the information coding and processing capabilities of the vLPO^GABA^ neurons during anesthesia and NREM sleep by analyzing the entropy, which reflects the randomness of population neural activity.^[^ ^59^ ^]^ We found that compared with wakefulness, the entropy significantly decreased during anesthesia but increased during NREM sleep (*Δ*_entropy_: −14.6%, 95% CIs [≈ −23.6% to −12.1%]; −11.7%, [≈ −14.6% to −9.1%]); −10.8%, [≈ −13.3% to −7.7%]); −13.1%, [≈ −15.7% to −10.8%]); and 3.4%, [≈1.1% to 6.1%] for PPF, ISO, KET, DEX, and NREM conditions, respectively) (Figure 4F), providing additional evidence to support the notion that anesthesia and NREM sleep induced different states of the vLPO neural activity.

### 2.5 Anesthesia and Sleep Evoke Divergent Modulation to the vLPO Glutamatergic Neurons

To further investigate whether the divergent modulations to the vLPO^GABA^ neural activity under anesthesia and sleep could also be applied to other cell types in the vLPO, we measured neural activity of the vLPO glutamatergic neurons (vLPO^Glut^) by carrying out micro‐endoscopic imaging in VGLUT2‐Cre mice.

We recorded 146–171 neurons from four mice under the anesthesia induced by the four anesthetics (DBI = 0.41 ± 0.09, 0.45 ± 0.06, 0.57 ± 0.11, and 0.23 ± 0.02, for PPF, ISO, KET, and DEX, respectively; mean ± s.e.m.). Similar to the vLPO^GABA^ neurons, the vLPO^Glut^ neurons exhibited diverse modulations in response to the application of various anesthetics, and the vast majority of them was suppressed during anesthesia (Figure S10A–D, Supporting Information)—averaged NMI: −0.99 ± 0.03, −0.75 ± 0.05, −0.66 ± 0.04, and −0.59 ± 0.05 (mean ± s.e.m.) for PPF, ISO, KET, and DEX, respectively; percentage of inhibited neurons (NMI < −0.33): 90%, 69%, 68%, and 62% for PPF, ISO, KET, and DEX, respectively—among which, PPF produced a predominant inhibition to the vLPO^Glut^ neurons, with > 90% of neurons showing NMI < −0.33 (Figure S10D–F, Supporting Information). Further analysis showed a large overlap between the neurons that were inhibited by each anesthetic—60 ± 1.9% of neurons inhibited by one anesthetic was also inhibited by another anesthetic (Figure S11H, Supporting Information).

Despite the overall inhibition of the vLPO^Glut^ neurons caused by these anesthetics, different anesthetics appeared to produce quantitatively different modulations to the same vLPO^Glut^ neuron, because there was no correlation between the NMI of individual neurons evoked by different anesthetics (the mean correlation coefficient for all pair‐wised comparisons equaled to 0.07 ± 0.05, mean ± s.e.m.) (Figure S11A–G, Supporting Information).

**Figure 5:**
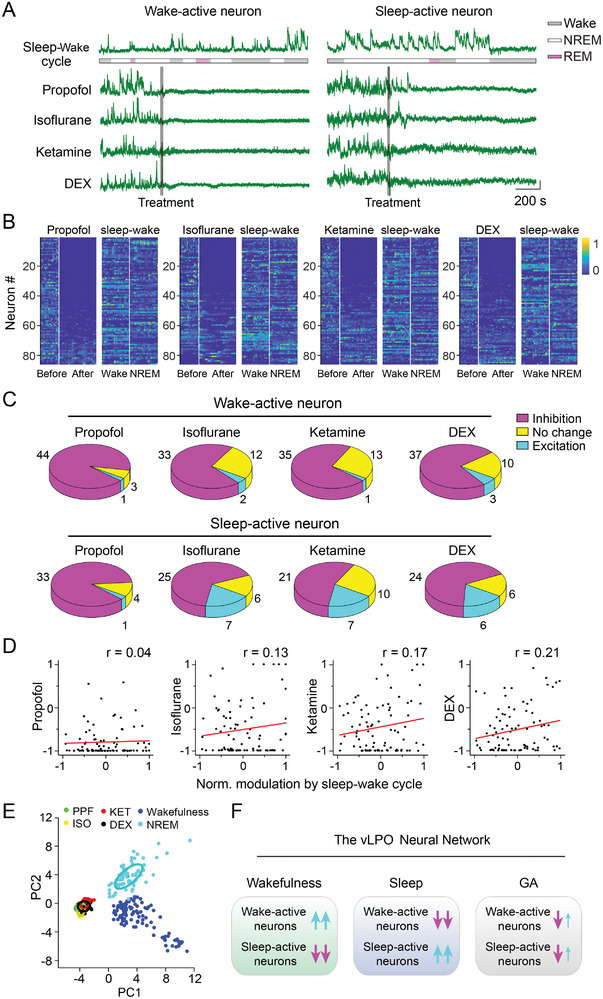
Anesthesia and sleep cause distinct modulation of the vLPO glutamatergic neurons. A–E) Same as Figure 4A–E, except that recording was made from the vLPO glutamatergic neurons. In (B,D), *n* = 86, 85, 87, and 86 for PPF, ISO, KET, and DEX, respectively. In (E), *n* = 68 neurons. F) A schematic diagram summarizing distinct states of the vLPO neurons in different conditions.

In the comparison between neural modulation under anesthesia and sleep, we identified 85 and 86 neurons from four mice that were recorded during all five experiment conditions, and found that different anesthetics produced similar inhibition to both sleep‐active neurons and wake‐active neurons (**Figure** **5**A–C)—68.6% (103 out 150) sleep‐active neurons were inhibited and 76.8% (149 out 194) wake‐active neurons were inhibited (Figure 5C), and there was little correlation between the modulation induced by anesthesia and sleep—the correlation coefficient ranged from 0.04 to 0.21 (Figure 5D). In the PCA analysis of vLPO^Glut^ neural activity, the four anesthetics also produced a distinct cluster with that during NREM sleep and wakefulness (Figure 5E and Figure S12, Supporting Information). In the entropy analysis, the vLPO^Glut^ activity showed more randomness during NREM sleep but less randomness during anesthesia (*Δ*_entropy_: −28.9%, 95% CIs [≈ −30.9% to −26.9%]; −13.2%, [≈ −16.2% to −8.6%]); −19.4%, [≈ −23.0% to −15.4%]); −21.7%, [≈ −25.1% to −18.5%]); and −0.61%, [≈ −3.86% to 2.69%] for PPF, ISO, KET, DEX, and NREM conditions, respectively) (Figure S13, Supporting Information). Together, these results suggested that anesthesia and sleep also produced different states of the vLPO^Glut^ neural activity.

### 2.6 Anesthetics Suppressed the vLPO Galaninergic Neurons

We have shown that anesthetics mainly inhibit both wake‐active and sleep‐active vLPO neurons, including GABAergic and glutamatergic neurons. However, the four anesthetics also activated a small group of sleep‐active neurons, and it is possible that these neurons are the galaninergic neurons, which are the best‐known sleep‐active neurons in the vLPO.^[^ ^47^, ^60^, ^61^ ^]^ We therefore next examined how the four anesthetics modulate the activity of vLPO galanin neurons (vLPO^Gal^).

**Figure 6:**
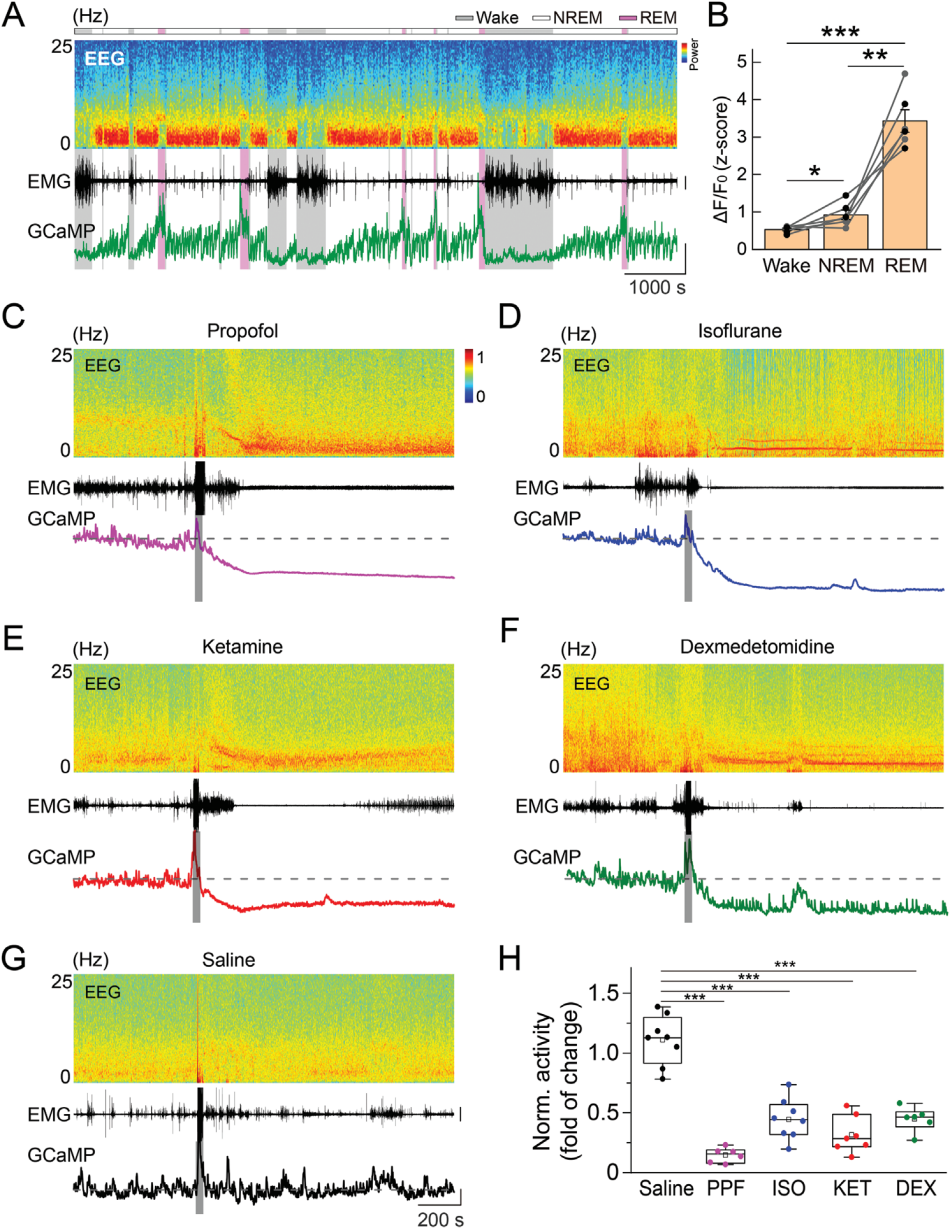
Anesthetics suppress the vLPO galanin neurons. A) Ca^2+^ activity of vLPO galanin neurons during the sleep‐wake cycle. Top to bottom: EEG power spectrogram; EMG (scale, 1 mV); photometry signals of GCaMP (scale, two z‐score). The brain states are color‐coded. B) GCaMP fluorescence in different brain states. Each line represents data from one recording. *n* = 6 sessions from three mice; Wake versus NREM: *p = 0.033; REM versus NREM: **p = 0.0012; Wake versus REM: ***p < 0.0001; Paired *t*‐test. C–G) Example recordings showing the change in the population Ca^2+^ signal and EEG/EMG after the application of each anesthetic or control, as indicated in each panel. Scale: EMG, 0.5 mV; GCaMP signal, five normalized z‐score, and 200 s. The gray bar indicates the timing of each treatment. The Dash line indicates the baseline. H) Statistical summary of changes in GCaMP signal after each treatment. The box plot shows a ≈25% to 75% range, the line shows a range within the 1.5 interquartile range (IQR), and the dot represents the median. *n* = 6, 8, 7, 6, and 8 sessions from three mice for PPF, ISO, KET, DEX, and saline, respectively. ***p < 0.0001 for all comparisons (comparing with the saline condition, one‐way ANOVA with post hoc Tukey's test).

We injected AAV expressing GCaMP6s into the vLPO of Gal‐Cre mice^[^ ^62^ ^]^ and measured the population Ca^2+^ signal using fiber photometry (**Figure** **6**A). The population Ca^2+^ signal rather than single‐cell imaging was used because the galanin neurons are a relatively homogenous group of sleep‐active neurons.^[^ ^47^, ^60^, ^61^, ^63^ ^]^ We first recorded the Ca^2+^ signal during the sleep‐wake cycle and found that galanin neurons were highly active during both NREM and REM sleep (Figure 6A,B), consistent with previous reports.^[^ ^47^, ^60^, ^61^ ^]^ However, the Ca^2+^ activity of the vLPO^Gal^ neurons was also significantly inhibited by the application of the four anesthetics (Figure 6C‐H) (*p* < 0.0001 for all four anesthetics, one‐way ANOVA with post hoc Tukey's test), further supporting that anesthesia and sleep may not recruit the same neural circuits.

Although all four anesthetics inhibited the vLPO^Gal^ neurons, we also noticed some rapid Ca^2+^ transients after the treatment with DEX (Figure 6F), suggesting a possible role for the galanin neurons in DEX‐induced anesthesia. This result is consistent with a recent finding that lesion of LPO galanin neurons attenuates the effects of DEX.^[^ ^64^ ^]^

### 2.7 Contributions of vLPO Neurons During Anesthesia

Our direct measurement of populational neural activity at a single neuron level during anesthesia and sleep showed that anesthetics caused different modulations of the vLPO activity than during sleep. These results strongly argue against the shared circuit hypothesis of anesthesia and sleep. However, multiple works that support this hypothesis have demonstrated overlapping neuronal activation under these two conditions and modulation of anesthesia by sleep circuits in the vLPO.

**Figure 7:**
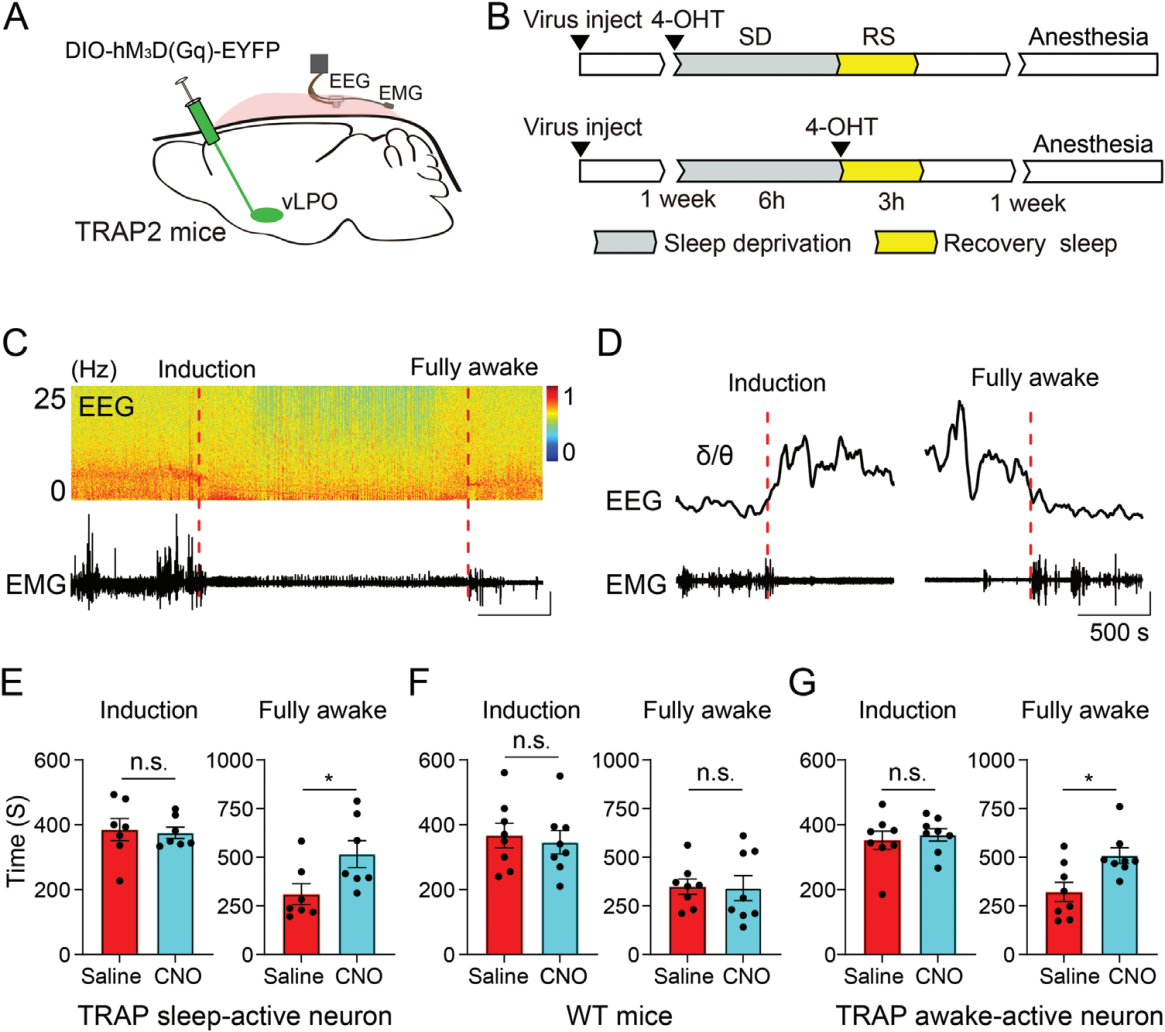
Contributions of vLPO neurons during anesthesia. A) Schematic of the experiment. B) Schematic of experimental design. TRAP2 mice were injected with AAV expressing hM3Dq one week before the 6‐h sleep deprivation (SD). For the “Wake TRAP” group, 4‐OHT was injected immediately before the SD, and for the “Sleep TRAP” group, 4‐OHT was injected immediately after the SD, before the recovery sleep. Effects on the isoflurane‐induced anesthesia were examined 1 week after the SD. C) Representative EEG and EMG recording during isoflurane (1.2%)‐induced anesthesia. Top, EEG spectrogram; Bottom, EMG, 0.5 mV, and 500 s. Dashed lines indicated the start and stop of the fully anesthetized time. D) Example of EEG *δ*/*θ* (top) and EMG (bottom) during the start (left) and stop (right) of the fully anesthetized state. Scale:0.5 mV, and 500 s. E) Statistical analysis of induction time and fully awake time for the “Sleep TRAP” group (induction time, *p* = 0.73, paired *t*‐test; fully awake time, *p = 0.016, Wilcoxon signed‐rank test; *n* = 7 mice). F) Statistical analysis of induction time and fully awake time for mice without expressing hM_3_Dq (induction time, *p* = 0.75; fully awake time, *p* = 0.92, *n* = 8 mice; paired *t*‐test). This experiment was used to control for nonspecific effects of CNO. G) Statistical analysis of induction time and fully awake time for the “Wake TRAP” group (induction time, *p* = 0.53, paired *t*‐test; fully awake time, *p = 0.023, Wilcoxon signed‐rank test; *n* = 8 mice).

To address the appeared inconsistency, we examined the modulation to anesthesia by sleep‐active vLPO neurons using the targeted recombination in active populations (TRAP) method^[^ ^65^ ^]^ (**Figure** **7**A). We first injected a viral vector expressing Cre‐dependent hM_3_Dq (a designer receptor exclusively activated by designer drugs, DREADDs)^[^ ^66^ ^]^ in the vLPO of the TRAP2 mice^[^ ^67^, ^68^ ^]^ (Figure 7B and Figure S14, Supporting Information). We then captured sleep‐active neurons one week later by intraperitoneal (i.p.) injection of 4 hydroxytamoxifen (4‐OHT) immediately before the recovery sleep after 6 h of sleep deprivation. This method can produce a specific expression of the hM_3_Dq in vLPO sleep‐active neurons.^[^ ^69^ ^]^ One week after tamoxifen induction, we injected (i.p.) the hM_3_Dq ligand clozapine‐N‐oxide (CNO) to activate the hM_3_Dq‐expressing neurons and examined their contribution to the anesthesia induced by ISO (Figure 7C,D). We found that CNO injection significantly increased the recovery time (Saline: 310 ± 54 s, CNO: 516 ± 69 s; mean ± s.e.m.; *p* = 0.016, Wilcoxon signed‐rank test) (Figure 7E), suggesting that sleep‐active vLPO neurons play a role in promoting anesthesia. This increase in recovery time was not a nonspecific effect of CNO, as CNO caused no detectable changes in mice that did not express hM_3_Dq (Saline: 348 ± 39 s, CNO: 341 ± 64 s; mean ± s.e.m.; *p* = 0.92, paired *t*‐test) (Figure 7F). This result is consistent with previous reports^[^ ^19^ ^]^ and appears to support the shared circuit hypothesis. However, this result has an alternative explanation—activation of the vLPO neurons may favor anesthesia, regardless of whether these neurons are sleep‐active or wake‐active.

To test the above possibility, we examined the contribution of the wake‐active vLPO neurons using the TRAP2 mice via a similar method (Figure 7B). Consistent with our prediction, we found that activation of the wake‐active vLPO neurons also increased the recovery time of the ISO‐induced anesthesia (Saline: 321 ± 50 s, CNO: 508 ± 40 s; mean ± s.e.m.; *p* = 0.023, Wilcoxon signed‐rank test) (Figure 7G), similar to the modulation caused by sleep‐active vLPO neurons. These results strongly suggest that different subpopulations of vLPO neurons can cause comparable modulation to drug‐induced anesthesia but are not necessarily related to their role in sleep‐wake regulation.

## 3 Discussion

In this study, we aimed to test a classical hypothesis that anesthesia and sleep engage the same neural ensemble and thus create similar brain states. As a first step towards this goal, we measured the population neural activity in one of the core sleep circuits—the vLPO^GABA^ neurons using fiber photometry recording and micro‐endoscopic Ca^2+^ imaging and systematically compared the neural activity of the same neuron during the natural sleep‐wake cycle and under anesthesia induced by four commonly used anesthetics or sedatives. We found that the four anesthetics caused both inhibition and excitation to the vLPO^GABA^ neurons (Figure 2), and different anesthetics appeared to have similar yet quantitatively different modulations to the same neuron (Figure 3). However, regardless of the diverse underlying mechanisms of the four anesthetics, GA‐induced modulation had little consistency with the neuron's firing pattern during the sleep‐wake cycle (Figure 4), suggesting that the state of the vLPO neural activity during anesthesia was different from that in natural sleep (Figure 5F). We also demonstrated that the divergent modulation of neural activity during anesthesia and sleep was also true for the vLPO glutamatergic neurons (Figure 5), further supporting that anesthesia and sleep cause different states of the vLPO neural activity. Finally, Our direct visualization of neural activity of the population single neuron in the state space using the PCA method illustrated divergent states of the vLPO^GABA^ neurons and vLPO^Glut^ neurons during wakefulness, sleep, and anesthesia. Collectively, these results showed that anesthesia and sleep caused different modulations of the vLPO neural population, suggesting that anesthesia and sleep may not engage the same neural circuits.

Our result is supported by recent work reporting that direct activation of the vLPO^GABA^ neurons fails to modulate anesthetic state transitions^[^ ^70^ ^]^ and is also consistent with previous studies showing that specific sleep‐regulating circuits may not be required for GA.^[^ ^71^, ^72^, ^73^ ^]^ Together, these studies provide evidence that argues against the shared circuit hypothesis for anesthesia and sleep.^[^ ^48^ ^]^

Various anesthetics can cause inhibition of global neural activity.^[^ ^74^, ^75^, ^76^ ^]^ Our results, obtained using fiber photometry recording the population Ca^2+^ signal from the vLPO neurons—including the GABAergic neurons, glutamatergic neurons, and the sleep‐active galanin neurons— clearly support the general inhibition hypothesis of the anesthesia.^[^ ^28^, ^30^, ^38^, ^53^ ^]^ However, recordings at the single‐neuron level showed that the four commonly used anesthetics caused both inhibition and excitation of the neural activity, which is consistent with studies using IEGs‐related methods (e.g., c‐Fos staining)^[^ ^19^, ^21^ ^]^ or electrophysiology^[^ ^21^, ^77^ ^]^ reporting that there are neurons being activated during GA.^[^ ^21^, ^29^, ^78^ ^]^ Despite the fact that different anesthetics have diverse acting mechanisms,^[^ ^8^, ^9^, ^11^, ^12^ ^]^ our longitudinal comparison showed that the four anesthetics tended to induce a similar modulation to the same vLPO neuron, although these modulations were often quantitatively different. In the four tested anesthetics, propofol and isoflurane mainly enhance GABAergic transmissions, and dexmedetomidine can inhibit alpha‐2 adrenergic receptor‐expressing neurons. Therefore, it is not surprising that they cause large inhibitory effects. However, we found that ketamine, which increases cortical activity, also caused a strong inhibitory effect on vLPO neurons. These findings suggested that the anesthesia‐modulated vLPO neurons may encode specific common features (e.g., vital signs) during anesthesia induced by different anesthetics.

Previous studies using IEGs‐related methods suggest an overlap between neurons activated during anesthesia and sleep.^[^ ^19^, ^21^, ^79^ ^]^ Our results showed that some vLPO^GABA^ neurons selectively active during sleep had increased activity after applying the four anesthetics, thus providing additional direct evidence supporting the above idea. However, our quantitative analysis of the activity in the same neurons under anesthesia and sleep also revealed a significant difference between the modulations caused by anesthesia and the sleep‐wake cycle. More importantly, we only recorded a limited portion of neurons that exhibited such correlated modulation; in contrast, most neurons being inhibited or activated during anesthesia seemed to have no significant correlation with their modulations during the sleep‐wake cycle. This lack of substantial overlap between the anesthesia‐active neurons and the sleep‐active neurons does not necessarily mean that the anesthesia‐active neurons can not affect the sleep‐wake regulation; indeed, elegant studies by selective activation of the anesthesia‐active neurons showed that these neurons had a notable contribution in the sleep‐wake regulation.^[^ ^16^, ^21^ ^]^ These results suggested an interaction of different brain circuits in the control of the sleep‐wake cycle.

On the other hand, with our current evidence, we cannot rule out the possibility that this small number of vLPO neurons that were active during both anesthesia and sleep may play a critical role during anesthesia. However, this does not affect our main conclusion that anesthesia induced a different state of the core sleep circuit than that during natural sleep. This is also the limitation of previous studies using IEG‐related methods^[^ ^15^, ^16^, ^17^, ^19^, ^20^, ^21^, ^79^ ^]^—neurons being captured by the expression of c‐Fos were not necessarily playing a role in driving anesthesia; they may be activated by the state of anesthesia. Indeed, in our longitudinal imaging of the same neuron's activity during anesthesia and sleep, we have clearly demonstrated that the majority of neurons that are active during anesthesia—theoretically, many of these neurons may be captured by IEG‐related methods, e.g., capturing activated neuronal ensemble (CANE), TRAP—do not even increase their activity during sleep and thus unlikely to play a role in driving sleep. Also, IEG‐related methods can only capture a static representation of the neural activity without knowing the neural dynamics that were offered by our longitudinal imaging experiments.

Our results from experiments using the TRAP2 mice showed that both vLPO neurons that were active during sleep or wakefulness could contribute to anesthesia similarly independent of their role in sleep‐wake regulation (Figure 7). This result raised an interesting alternative explanation for previous work that is in favor of the shared circuit hypothesis^[^ ^1^, ^11^, ^16^, ^64^, ^80^ ^]^—the experimental effects might be attributed to the activation of a subset of neurons in the vLPO, while not because these neurons are specifically active during sleep or anesthesia; activation of other subpopulations may cause similar modulations. This alternative explanation raised concerns about evidence obtained using the IEG‐related methods and explained the conflict between studies supporting or against the shared circuit hypothesis.

It should be noted that the four anesthetics or sedatives tested in our study cause different levels of anesthesia, ranging from sedation to surgical anesthesia, primarily due to the distinct characteristics of each drug. However, in these widely different brain states induced by these drugs, we observed no apparent consistency between their modulations to both GABAergic neurons and glutamatergic neurons in the vLPO and the modulations during sleep, further supporting our conclusion that anesthesia and sleep are different brain states. We would also like to note that since circadian factors can have a significant modulating effect on anesthesia states,^[^ ^81^ ^]^ experiments performed during different circadian phases (e.g., during the dark phase) may have yielded different results.

## 4 Conclusion

In summary, we have provided a direct comparison between the neural activity of the same group of single‐neuron during anesthesia and natural sleep. Our results suggest that anesthesia may not recruit the same neural circuits involved in sleep‐wake regulation in an identical manner. Given the brain‐wide distribution of receptors that are targeted by anesthetic agents, studying discrete brain regions does not give a network picture of what is happening under general anesthesia, and is unlikely to explain how it produces unconsciousness. Thus, future studies measuring global neural activity (e.g., macroscopic Ca^2+^ imaging) are needed to understand the network mechanism of anesthetic‐induced unconsciousness.

## 5 Experimental Section

### Animals

All experimental procedures followed the National Institutes of Health guidelines and were approved by the Animal Care and Use Committee at the Institute of Neuroscience, Chinese Academy of Sciences. Adult VGAT‐IRES‐Cre (Jackson #: 016962), VGLUT2‐IRES‐Cre (Jackson #:016963), and Gal‐Cre (MMRRC #:036969‐UCD) mice (male, > 8 weeks at the time of surgery) were used for experiments. Mice were housed in a 12/12‐h light/dark cycle (light on at 7 am) with food and water available ad libitum. Mice with implants for EEG/EMG recordings, fiber photometry, or micro‐endoscope imaging were housed individually.

### Surgery

Mice were anesthetized with isoflurane (5% for induction, 1.5% for maintenance) and placed on a stereotaxic frame with a heating pad. After shaving the hair and cleaning the incision site with iodine and medical alcohol, the scalp was incised to expose the skull. A small craniotomy (≈1 mm in diameter) was drilled for virus injection, optical fiber, or GRIN lens implantation. To monitor the Ca^2+^ activity of GABAergic or glutamatergic neurons in the vLPO, a Cre‐dependent adeno‐associated virus expressing Ca^2+^ indicator, rAAV‐Ef1*α*‐DIO‐Gcamp6s‐WPRE‐pA (BrainVTA, #PT‐0071, 5.4 × 10^12^ vg ml^−1^, 200 nl), was injected into the vLPO (AP: 0, ML: 0.7, DV: 5.0; Injected with no angle). For chemogenetic experiments using the TRAP2 mice, hM_3_Dq (rAAV‐Ef1*α*‐DIO‐hM_3_Dq‐EYFP‐WPRE‐pA, BrainVTA, #PT‐0816, 2.8 × 10^12^ vg ml^−1^, 150 nl) were bilaterally injected into the vLPO.

For fiber photometry recording, an optical fiber (200 µm, 0.37 NA) with an FC ferrule was carefully inserted into the same coordinate used for virus injection. For micro‐endoscope imaging, a GRIN lens (0.5 mm in diameter, Gofoton #: GFK‐000224‐PO) was implanted as described previously.^[^ ^56^ ^]^ Briefly, a 0.5 mm diameter optical fiber with a sharpened tip was inserted to create a tunnel for lens insertion, and then the lens was pushed down to ≈200 µm above the target brain area and secured to the skull with dental cement. A piece of PCR tube was used as a protective cap to cover the GRIN lens.

For EEG/EMG recording, two stainless steel screws for EEG were inserted into the skull above the visual cortex and the frontal cortex, two insulated EMG electrodes were inserted into the neck muscle, and a reference electrode was attached to a screw inserted into the skull above the cerebellum.

All implants were secured to the skull with dental cement, and experiments were carried out at least one week after surgery.

### TRAP Induction

To selectively manipulate neurons that were active during wakefulness or sleep, the TRAP2 mice were used, using the same procedure described previously.^[^ ^69^ ^]^ Mice were subjected to 6‐h sleep deprivation (SD), starting at the beginning of the light period (7 am), and for the “Wake‐TRAP” group, 4‐OHT was injected at the start of the SD. For the “Sleep‐TRAP” group, 4‐OHT was injected at the end of the SD, immediately before the recovery sleep. The SD was achieved by introducing novel objects or tapping lightly on the cage. To reduce the possibility of stress, the mice were not directly touched. The same dose of 4‐OHT (40 mg kg^−1^) was used as reported previously.^[^ ^69^ ^]^

### Induction of Anesthesia

To induce anesthesia using the four anesthetics or sedatives, doses that were reported previously to induce loss of consciousness (propofol, 180 mg kg^−1^, i.p.; ketamine, 100 mg kg^−1^, i.p.; dexmedetomidine, 100–150 µg kg^−1^, i.p.; isofluorane, induction, 2.5% (v/v), maintenance 1% (v/v) were used.^[^ ^16^, ^21^, ^32^ ^]^ Each session consisted of 10 min baseline recording and a recording of ≈20++40 min during anesthesia. Mice that received multiple treatments were allowed to rest for 48–72 h. The order of injections was PPF, ISO, KET, DEX, and saline.

### Polysomnography Recording

Mice were connected to flexible recording cables via a mini‐connector to record EEG/EMG signals. For polysomnography recordings during fiber photometry experiments, recordings were performed in the home cage. For polysomnography recordings during micro‐endoscope imaging, experiments were carried out on head‐fixed mice after habituating them to the recording apparatus. The EEG/EMG signals were recorded using TDT system‐3 amplifiers (RZ2 + PZ5) with a high‐pass filter at 0.5 Hz and digitized at 1500 Hz. All the experiments started at 1 pm.

### Fiber Photometry Recording

To record fluorescence from the GCaMP6s, an optic fiber (Thorlabs, FT200UMT) was attached to the implanted ferrule via a ceramic sleeve, and emission fluorescence was recorded using a customized fiber photometry setup described previously.^[^ ^82^ ^]^ The photometry rig was constructed using parts from Doric Lens, including a fluorescence mini cube (FMC4_AE(405)_E(460‐490)_F(500‐550)_S), a blue LED (CLED_465), a LED driver (LED_2), and a photoreceiver (NPM_2151_FOA_FC). A software‐controlled lock‐in detection algorithm was implemented during recording in the TDT RZ2 system using the fiber photometry “Gizmo” of the Synapse software (modulation frequency: 459 Hz; low‐pass filter for demodulated signal: 20 Hz, 6th order). The intensity of the excitation light was measured as 10–20 µw from the tip of the optical fiber. The photometry data was recorded using a sampling frequency of 1017 Hz. To minimize the optical fiber's auto‐fluorescence, the recording fiber was bleached before each recording. The background autofluorescence before each recording was subtracted from the recorded signal in subsequent analysis.

### Micro‐Endoscope Imaging

Mice were habituated to the head‐fixed apparatus before imaging. A customized epifluorescence microscope was used to image the Ca^2+^ activity through a GRIN lens. The microscope was constructed using parts from Thorlabs, including an objective (Olympus, RMS10X‐PF), a GFP filter set, an excitation LED (470 nm), a LED driver, and a CCD camera (Qimaging, Retiga R1). The image was acquired using Micro‐manager software, with an acquisition rate of 5 Hz. EEG/EMG was recorded using a TDT system‐3 amplifier controlled by OpenEx software (TDT). Image acquisition and EEG/EMG were synchronized using an Adruino board. During the image acquisition process after anesthesia, towels or warm water pads were used to reduce the mice's body temperature drop.

### Chemogenetic Manipulation

For chemogenetic activation experiments, mice were first habituated to the recording chamber, saline (0.9% NaCl) or CNO (3 mg kg^−1^, in saline)^[^ ^24^ ^]^ was injected intraperitoneally (i.p.) 30 min before each test. During the test, mice were placed in a gas‐tight acrylic chamber, connected to EEG/EMG recording cable, and anesthetized using isoflurane (1.2%, v/v) for 30 min. During the experiment, the temperature at the bottom of the box was kept between 37 and 38 °C. Each mouse was subjected to three tests (interval between tests > 72 h).

EMG was used to determine the state of the anesthesia. The EMG power was first determined during anesthesia by computing the root‐mean‐square (RMS) values every 20‐s during the 20‐min before the stop of the isoflurane. A threshold was defined as mean + 3*SD of the RMS. The start of “fully anesthetized” was defined as the time that the EMG power was smaller than the threshold in 15 successive sliding windows (duration, 20 s with 10 s overlap). The time of “fully awake” was defined as the time that the EMG power was larger than the threshold in 9 windows. The algorithm's results were similar to those determined by trained experts during visual inspection of EEG and EMG and were consistent with previously reported results.^[^ ^21^ ^]^

### Histology

To verify the expression of GCaMP and placements of the optical fibers or GRIN lens, The brain tissues were processed according to procedures described previously.^[^ ^56^ ^]^ For GFP immunostaining, brain sections were permeabilized using PBST (0.3% Triton X‐100 in PBS) for 30 min and incubated with a blocking solution for 1 h before incubating with a primary antibody (GFP‐1020, AVES; 1:1000) overnight at 4 °C. The brain sections were then washed with PBS and incubated with a secondary antibody. Finally, the brain sections were washed with PBS and mounted with mounting media.

The fluorescence images were captured using an epifluorescence microscope (VS120, Olympus) or a confocal microscope (Eclipse Ni‐E or Ti‐E, Nikon).

### Polysomnography Analysis

To classify the brain states using EEG/EMG signals, a fast Fourier transform spectral analysis with a frequency resolution of 0.18 Hz was used. The brain states were scored every 5 s semi‐automatically using a MATLAB GUI and validated manually by trained experimenters. Brain states classification was performed according to established criteria:^[^ ^82^, ^83^ ^]^ Wakefulness was defined as desynchronized EEG and high EMG activity; NREM sleep was defined as synchronized EEG with high‐amplitude delta activity (0.5–4 Hz) and low EMG activity; REM sleep was defined as high power at theta frequencies (6–10 Hz) and low EMG activity.

### Fiber Photometry Analysis

To analyze the photometry data, the autofluorescence was first subtracted from the raw data and binned the signal into 1 Hz. The *ΔF*/*F*_0_ was calculated using a baseline obtained by fitting the autofluorescence‐subtracted data during baseline recording with a second‐order exponential function or using an adaptive baseline by a moving average of 100 s. To quantify the change in the GCaMP signal across multiple conditions/animals, the z‐score transformed *ΔF*/*F*_0_ was normalized using the standard deviation of the GCaMP signal during baseline and then used the SD of the signal after treatment to calculate the fold of change (Figures 1I and 6H).

### Micro‐Endoscope Imaging Analysis

Imaging data were processed in MATLAB (MathWorks). The motion correction using the rigid‐affine algorithm in ANTs (http://stnava.github.io/ANTs/) was first performed, and the Ca^2+^ signals were extracted using CNMF‐E (https://github.com/zhoupc/CNMF_E). The Ca^2+^ signal was normalized to the global background estimated in CNMF‐E and used for further analysis.

### Evaluating Depth of Anesthesia Using EEG/EMG Signals

EMG power and the ratio between EEG *δ* power (≈0.5–4 Hz) and *θ* power (≈6–10 Hz) were calculated every 5 s. A baseline (A recording period of 5‐min with active EMG) was manually selected, and a 10‐min sliding window after each treatment was used to generate the brain state cluster during anesthesia. A clustering analysis was then performed using the K‐means algorithm (unsupervised) and calculated Davies–Bouldin index (DBI) using the built‐in functions in MATLAB:

(1) $$\frac{1}{N}\sum_{i=1}^{N}\max_{j\neq i}\left(\frac{d^{\prime }\left(i\right)+d^{\prime }\left(j\right)}{d(i,j)}\right)$$

The DBI is a ratio between the intra‐cluster distances and the inter‐cluster distances. A smaller value meant better separation of the two clusters that represent the EEG/EMG during wakefulness and anesthesia, respectively. The time window was shifted every 1‐min to find a small DBI to determine the recording period with adequate depth of anesthesia.

To monitor the behavior of mice during anesthesia, an IR camera was used to capture the faces of the mice and also used the FaceMap software (www.github.com/MouseLand/FaceMap) to extract the facial movements.

The normalized modulation index (NMI) was defined as the Ca^2+^ signal (area under curve, AUC) after each treatment minus the Ca^2+^ signal during the baseline period divided by the sum of the two signals. Thus, the range of the NMI will be ≈ −1 to1, and neurons inhibited by anesthetics will have a negative NMI.

### Hierarchical Clustering Analysis

Hierarchical clustering (unsupervised) was used to classify response patterns of vLPO neurons to the applications of the four anesthetics. The Euclidean distance was calculated according to the NMI of neurons, and the agglomerative hierarchical cluster tree was generated using the “average” method.

### State Space Analysis

Principal component analysis (PCA) was used to construct a 2D state space of neural activity of the vLPO^GABA^ and vLPO^Glut^ neurons under various conditions. A total of 123 neurons (from 10 mice) were imaged for vLPO^GABA^ and 68 neurons (from four mice) for vLPO^Glut^ during the applications of all four anesthetics and the sleep‐wake cycle. Z‐score transformed Ca^2+^ signals with a 15‐s bin were used to calculate the state of the vLPO neurons. Gaussian mixture distribution with one component was fitted to each state, and the center and standard error were shown. To further validate the distinct states observed under each condition, a bin size of 30 s was used to repeat the analysis and generate (Figures S9 and S12, Supporting Information).

### Entropy Analysis

To estimate the change in the randomness of neuron activity caused by anesthesia and NREM sleep, the network entropy was calculated using population Ca^2+^ signals. For each condition (PPF, ISO, KET, DEX, and NREM), a 2‐min recording window was randomly sampled from baseline (wakefulness) and anesthesia or NREM period, the mean activity of each neuron during each 5‐s bin was defined as *S*_i,_ and the activation probability *P*_i_ of the *i*‐th 5‐s bin was calculated:

(2) $$p_{i}=S_{i}/\sum_{i=1}^{n}\mathrm{Si}$$

The entropy of each neuron during the 2‐min window was computed using Shannon's formula:^[^ ^84^ ^]^

(3) $$H\left(X\right)=-\sum_{i=1}^{n}p_{i}\log\;p_{i}$$

The mean entropy (H) of all neurons recorded under the condition was calculated to get Δ_entropy_ (H_anesthesia_ – H_baseline_).

To estimate the distribution of Δ_entropy_, we repeated the above procedure 5000 times and calculated the 95% confidence interval.

### Statistical Analysis

Statistical analysis was performed using MATLAB or OriginLab. All statistical tests were two‐sided. A normality test was first performed on each dataset using the Shapiro–Wilk test. Parametric tests (paired or unpaired Student's *t*‐tests) were used if the dataset was normally distributed (*p* < 0.05), otherwise non‐parametric tests (Wilcoxon signed‐rank test or Wilcoxon rank‐sum test) were used. All the statistical tests were two‐tailed and performed in MATLAB. The significance level was set at *p* = 0.05.

A calculation was not performed on the sample size. A sample size comparable to studies using similar techniques and animal models was used. For the fiber photometry and micro‐endoscope imaging experiments, the outlier sessions in which mice did not respond well to the treatments (with too many movements after injection) were removed.

The investigators were not blinded to the experimental conditions of the animals.

## Conflict of Interest

The authors declare no conflict of interest.

## Author Contributions

M.L. and X.F. contributed equally to this work. M.X. conceived the project and designed experiments. M.L. conducted the experiments with an assistant from X.L. and K.J. X.F. and M.L. analyzed the data. All authors contributed to data interpretation. M.X. wrote the paper with input from all authors. M.X. and Y.W. supervised all aspects of the work.

## Supporting information

Supporting Information

Supplemental Movie 1

Supplemental Movie 2

## Data Availability

The data that support the findings of this study are available from the corresponding author upon reasonable request.
