# Supplementary material for: Divergent Neural Activity in the VLPO During Anesthesia and Sleep
Source: Adv Sci (Weinh). 2022 Dec 3;10(2):2203395. doi: 10.1002/advs.202203395 (PMC9839870; doi:10.1002/advs.202203395)
Supplement: Supplementary file 1 — Supporting Information [file ADVS-10-2203395-s002.pdf]

ADVANCED SCIENCE
Open Access

Supporting Information

for *Adv. Sci.*, DOI 10.1002/advs.202203395

Divergent Neural Activity in the VLPO During Anesthesia and Sleep

Mengqiang Luo, Xiang Fei, Xiaotong Liu, Zikang Jin, Yingwei Wang and Min Xu**

Supporting Information

Divergent Neural Activity in the VLPO during Anesthesia and Sleep

Mengqiang Luo[1,4], Xiang Fei[2,4], Xiaotong Liu[2], Zikang Jin[2], Yingwei Wang[1,]*, and Min Xu[2,3]*

*Corresponding author. Email: wangyw@fudan.edu.cn (Y.W); mxu@ion.ac.cn (M.X)

This PDF file includes:

Supplementary Text

Figures S1 to S14

Other Supporting Information for this manuscript includes the following:

Movies S1 to S2

Supporting Information

Supporting Information includes 14 Figures and two movies.

Movie S1: An example showing changes in brain state during the application of DEX. The state space was defined by EMG power and EEG δ/θ.

a 2Ǿ⁄Ŝ {нΥ An example of Micro-endoscopic Ca^{2+} imaging of $VLPO^{GABA}$ neurons in response to the application of DEX. Videos showing the raw image, image after motion correction, and the de-noised image with 30x speed.

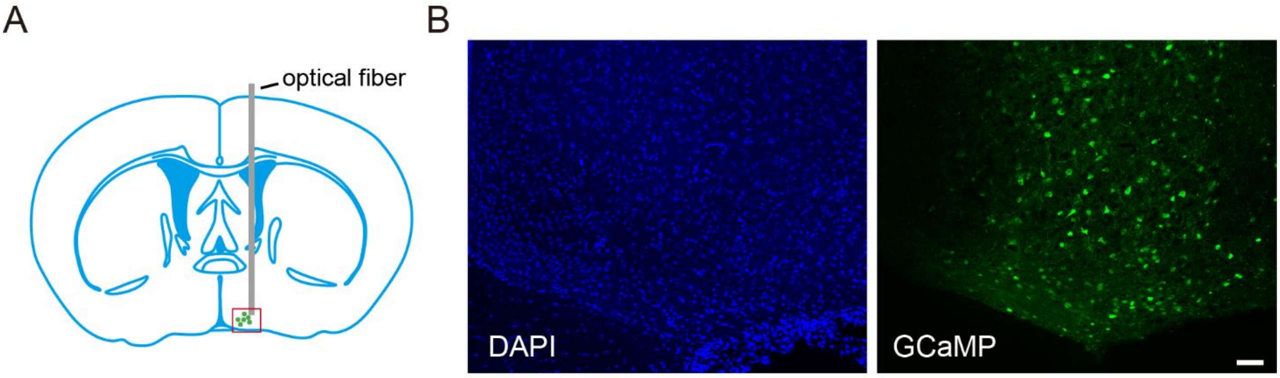

Figure S1. Histological verification of optical fiber position and virus expression.

A) Schematic diagram summarizing placement of the optic fiber in the VGAT-Cre mice expressing DIO-GCaMP6s in the vLPO. **B**) Images showing the virus expression. AAV expressing Cre-dependent DIO-GCaMP6s (rAAV-Ef1α-DIO-GCaMP6s-WPRE-pA) was injected into the vLPO. The scale bar represents 50 µm.

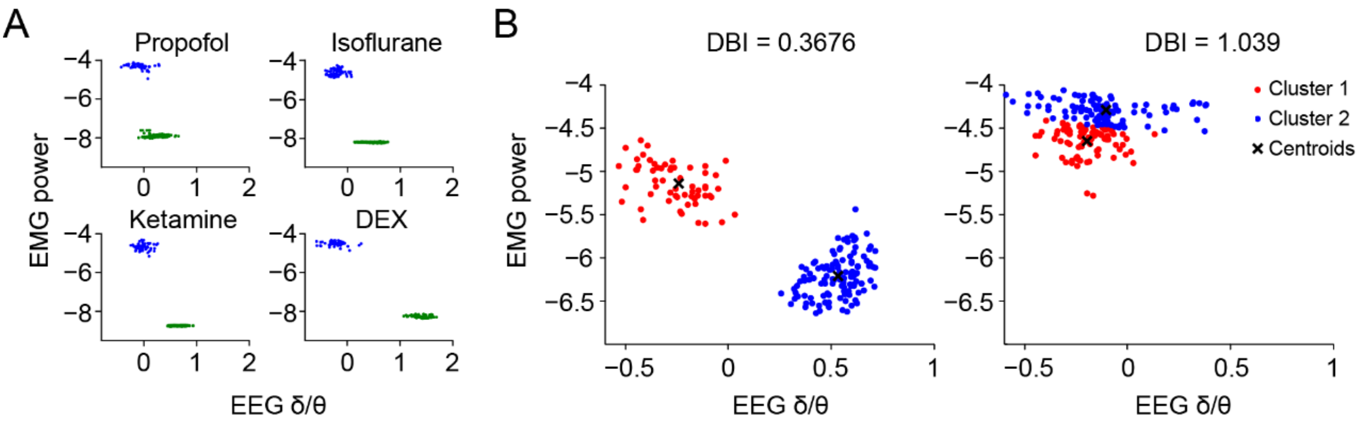

Figure S2. Modulations of EEG/MEG by anesthetics.

A) Examples showing EEG δ/θ and EMG before and after the application of each anesthetic. Axis is shown in the lg10 scale. Each dot represents the EEG δ/θ and EMG power in a 5-s bin. **B**) Examples illustrating the separation of two clusters. A smaller DBI means a good separation (e.g., the example in the left panel).

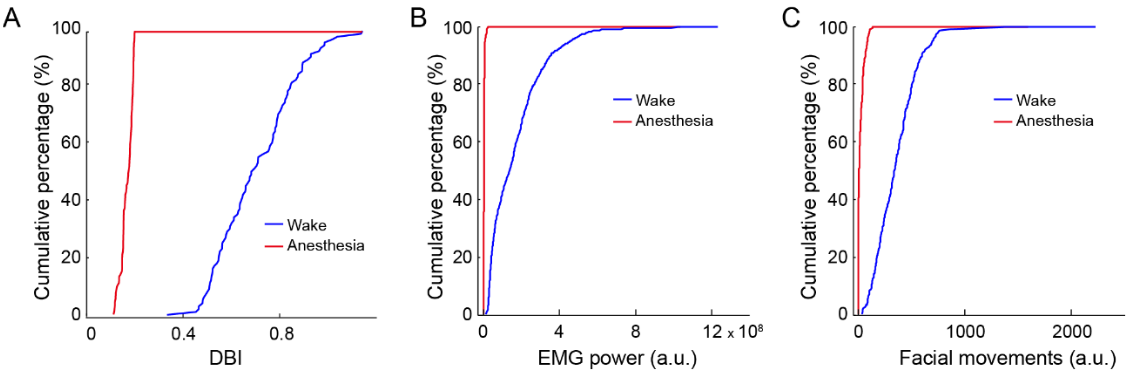

Figure S3. Use DBI to determine anesthesia states.

A) Cumulative distribution of DBI during wakefulness and anesthesia. Data were from 5 recordings from 3 mice. **B**) Cumulative distribution of EMG during wakefulness and anesthesia. Data were from 5 recordings from 3 mice. **C**) Cumulative distribution of facial movements during wakefulness and anesthesia. Data were from 5 recordings from 3 mice injected with ketamine or propofol.

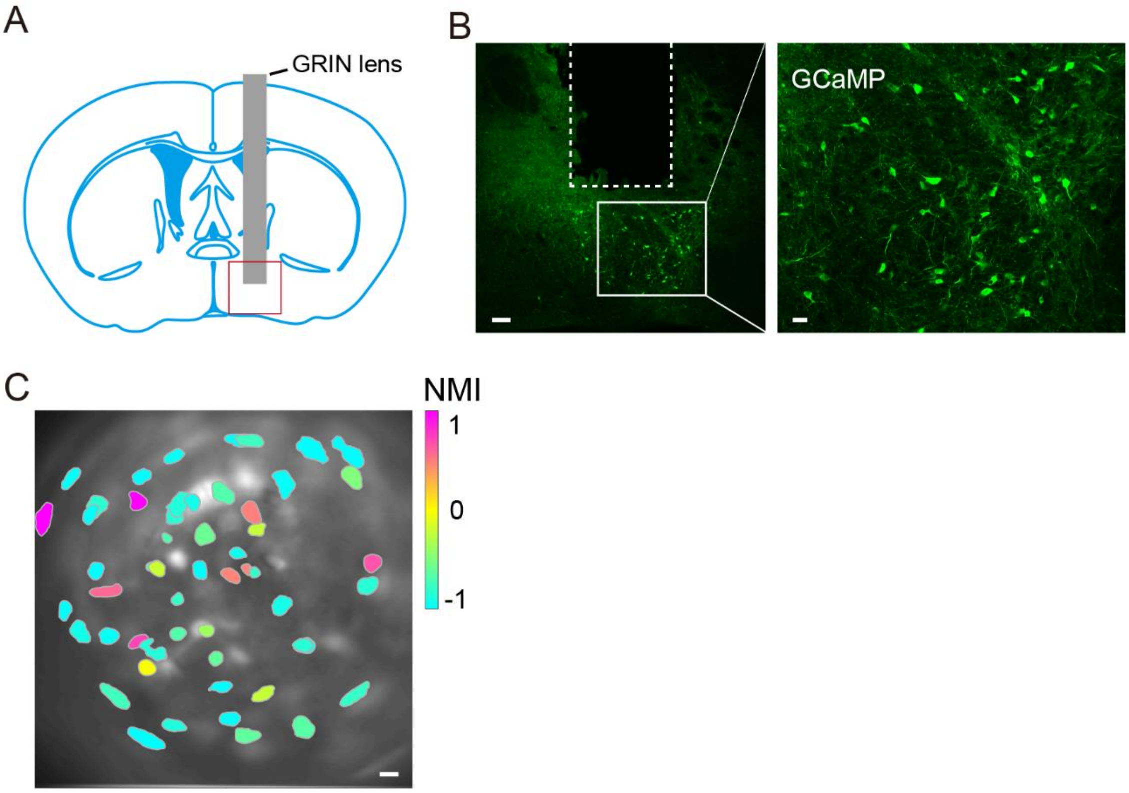

Figure S4. Micro-endoscopic imaging of GCaMP6s-expressing GABAergic neurons in the vLPO.

A) Schematic diagram summarizing placement of micro-endoscopic Ca^{2+} imaging through GRIN lens in the VGAT-Cre mice expressing DIO-GCaMP6s in the vLPO. **B**) Images showing the virus expression. AAV expressing Cre-dependent DIO-GCaMP6s was injected into the vLPO. The scale bar represents 100 μm (Left) and 25 μm (Right). **C**) Example regions of interest (ROI) in responding to the application of anesthetics. NMI of each neuron was color-coded. The scale bar represents 25 μm.

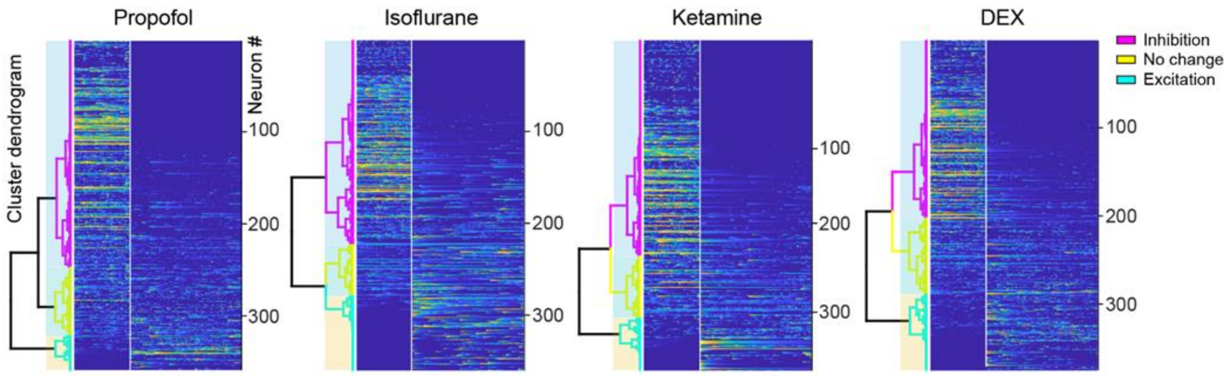

Figure S5. Classification of response patterns of vLPO^{GABA} neurons during anesthesia using hierarchical clustering method.

Cluster dendrogram of vLPO^{GABA} neurons' response to the indicated anesthetic. The heatmaps were the same as those in Figure 2E. Neurons were sorted by the NMI. Neurons in the three clusters were inhibited, excited, or insensitive to the application of anesthetics, respectively.

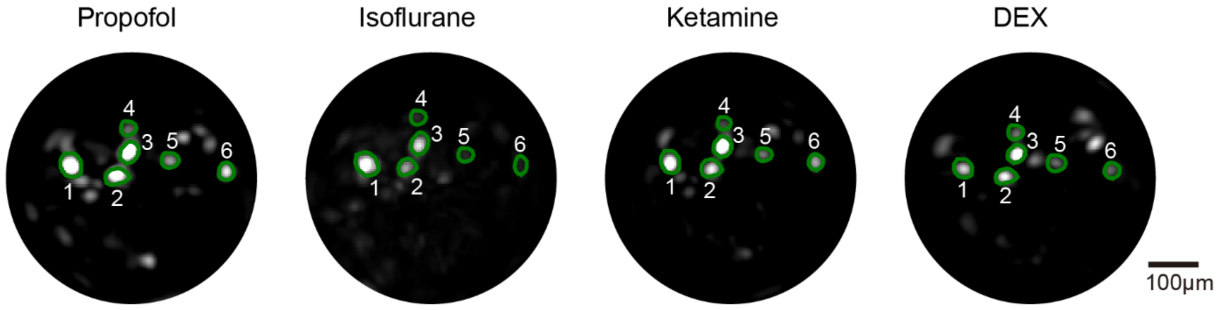

Figure S6. Longitudinal imaging of the same neural population across different conditions.

Example field of view after manually aligning the imaging data in each indicated condition. ROIs were outlined in green. The scale bar represents 100 μm.

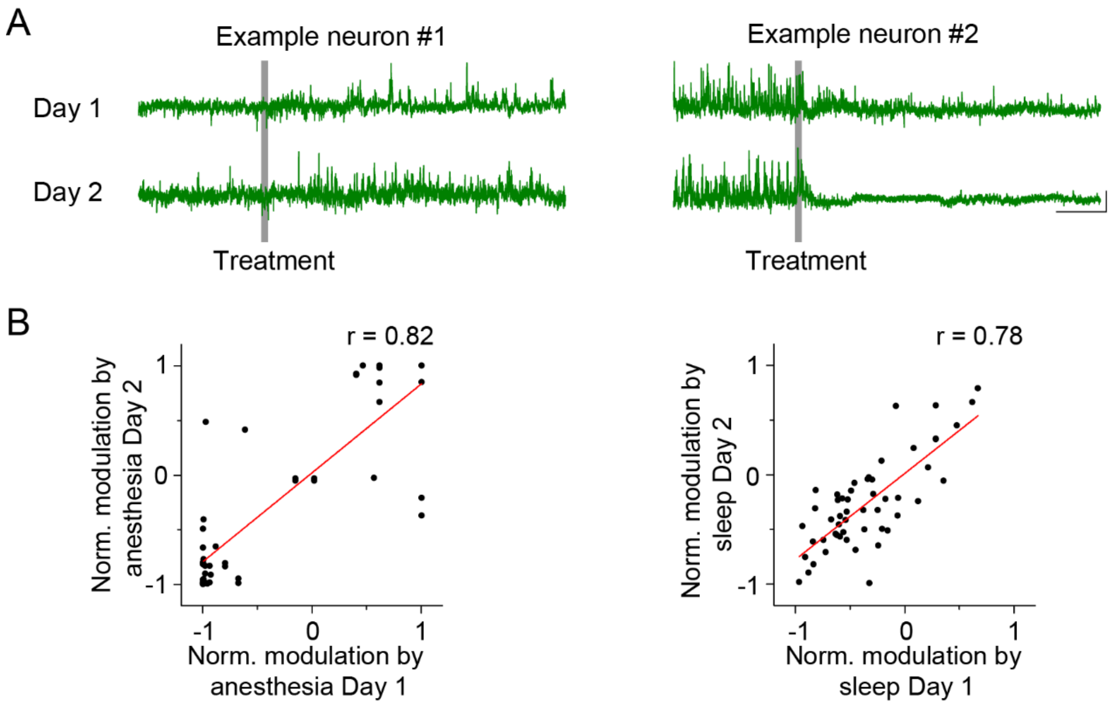

Figure S7. Individual vLPO GABAergic neuron shows a similar response to the same treatment during different experimental repeat.

A) The Ca^{2+} signals of two example neurons in response to the application of DEX on different days. The gray bar indicates the timing of the treatment; Scale bar, 10% ($\Delta F/F_0$) and 200 s. **B**) Scatter plot showing the correlation between the modulation evoked by different repeats of the same treatment (left, PPF; right, sleep-wake cycle). Each dot is one neuron. The red line is a linear fit of the data.

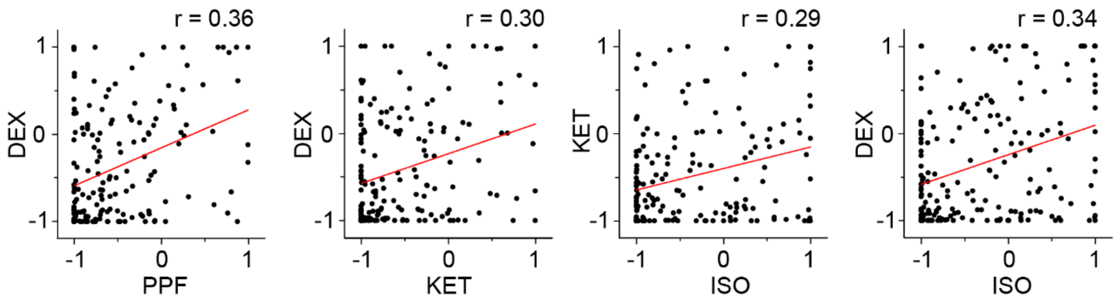

Figure S8. Different anesthetics engage similar vLPO GABAergic neural populations.

Scatter plot showing the correlation between the modulations evoked by indicated conditions (*n* = 176, 182, 175, and 197 for each condition).

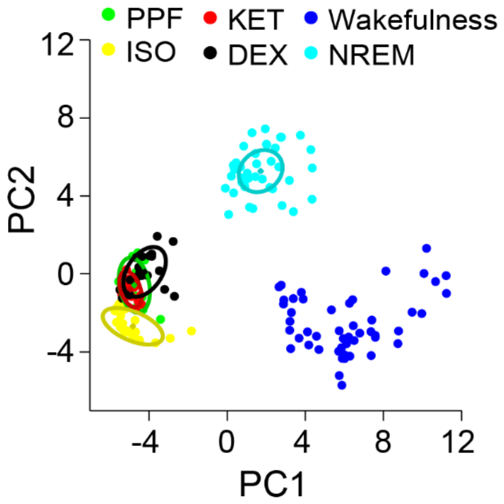

Figure S9. Anesthesia and sleep cause a distinct state of the vLPO GABAergic neurons.

Visualization of the neural activity in the state space using PCA. Neural activity under various conditions was color-coded. The plot was constructed using data from 123 neurons that were recorded in all five conditions; each dot represents population neural activity in a 30-s bin.

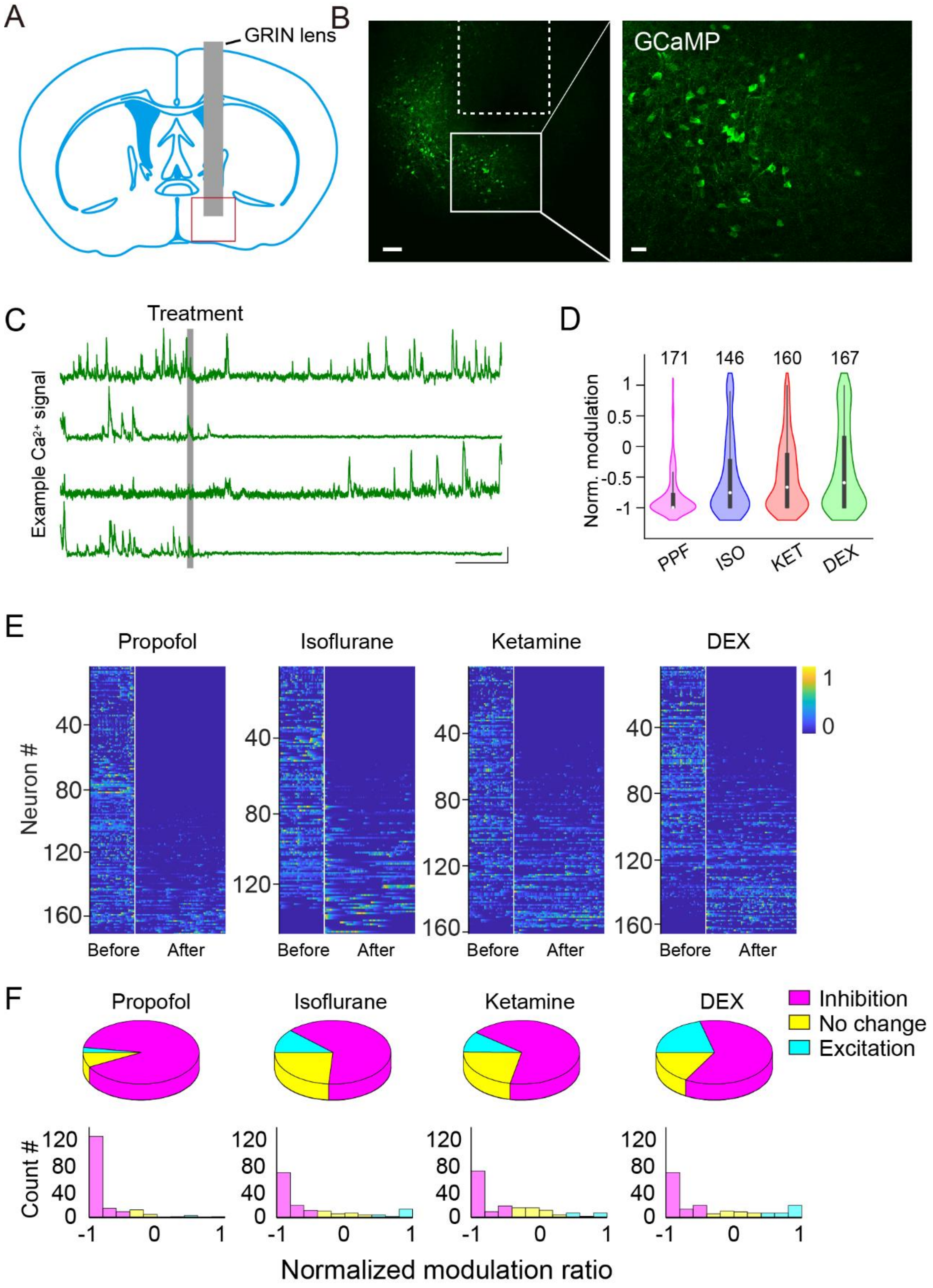

Figure S10. Anesthetics induce diverse modulation of Ca^{2+} signals to the glutamatergic neurons in the vLPO.

A) Schematic diagram depicting micro-endoscopic recording of population Ca^{2+} signal of glutamatergic neurons in the vLPO. The GCaMP6s-expressing neurons were imaged using an epifluorescence microscope via an implanted GRIN lens. **B**) Images showing the virus expression. AAV expressing Cre-dependent DIO-GCaMP6s was injected into the vLPO. Scale bar represents 100 µm (Left) and 25 µm (Right). C) Example Ca^{2+} signal (raw trace) from four simultaneously recorded neurons responding to the application of anesthetics. Each line is one neuron. The gray bar indicates the timing of the treatment. Scale bar, 10% ($\Delta F/F_0$) and 200 s. **D**) Statistical summary of the normalized modulation by each anesthetic. The violin plots were a combination of KDE and box plot. The box plot shows a 25%~75% range, the line shows a range within 1.5 IQR, and the dot represents the median. n = 171, 146, 160, and 167 for PPF, ISO, KET, and DEX, respectively. $p < 0.0001$ for all groups (Wilcoxon signed-rank test). **E**) Heat map showing the Ca^{2+} signal of each neuron during baseline and the period with an adequate level of anesthesia (determined using the DBI measurement). The recording periods were spliced together, and the white line indicates the splicing point. Neurons were sorted by the NMI. **F**) Pie chart showing the percentage of neurons modulated by each anesthetic (up). Distribution of the NMI induced by each treatment (low).

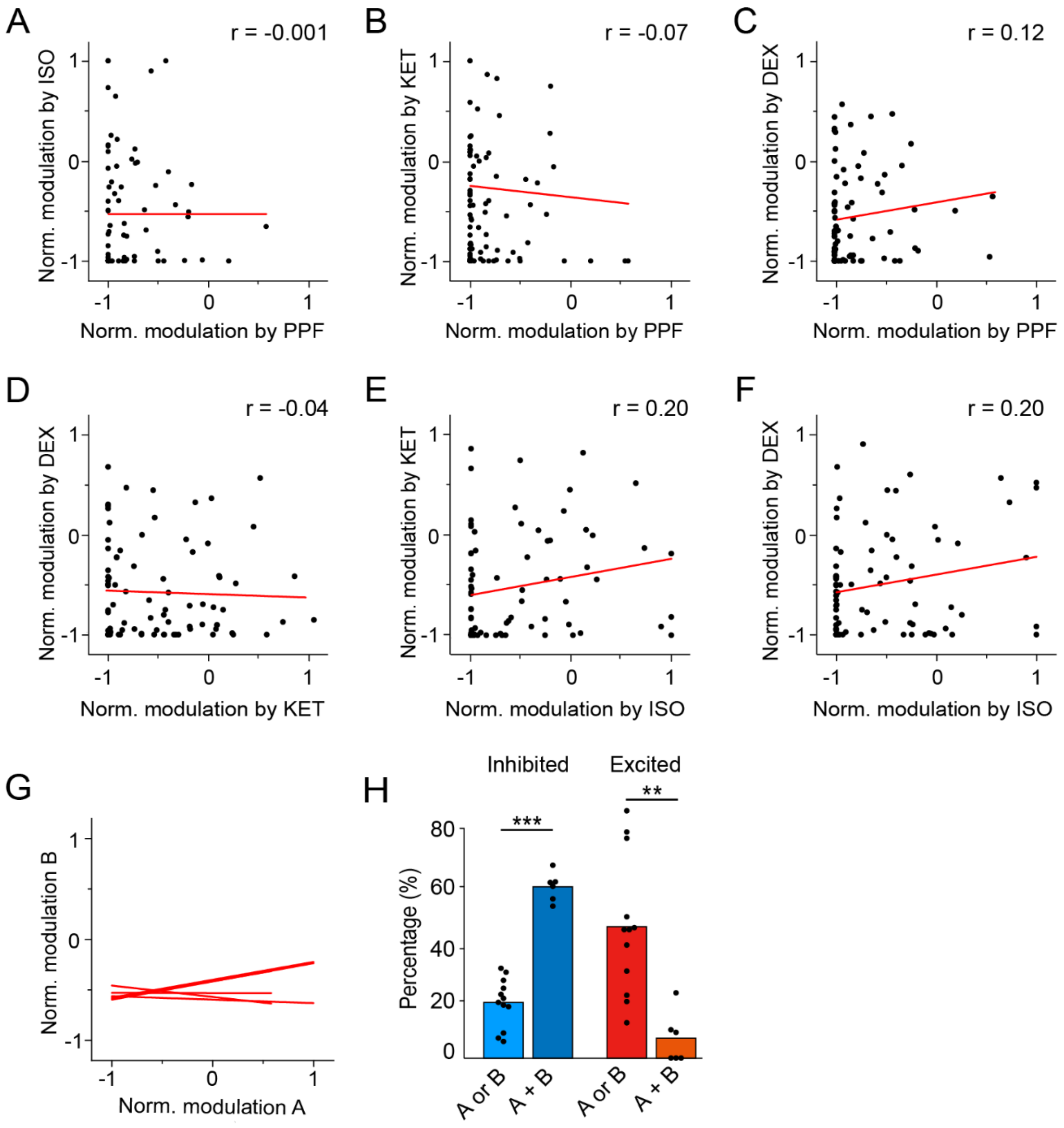

Figure S11. Different anesthetics engage similar vLPO glutamatergic neural populations.

A) Scatter plot showing the correlation between the modulation evoked by PPF and ISO (n = 80). Each dot is one neuron. The red line is the linear fit of the data. **B–F**) Same as A), except that the comparison was between the two indicated conditions. n = 89, 88, 87, 78, and 81 for B, C, D, E, and F, respectively. **G**) A summary showing the linear fit of all pairwise comparisons. H) Statistical summary for the percentage of neurons showing the same or different modulation by various anesthetics. Both inhibited 60.6 ± 1.9%, only inhibited by one anesthetic 19.7 ± 2.4%; both excited 7.0± 3.4%, only excited by one anesthetic 46.5 ± 6.7%, mean ± sem. ***p < 0.0001 and **p = 0.0016 for inhibited and excited group, respectively (Student's t-test).

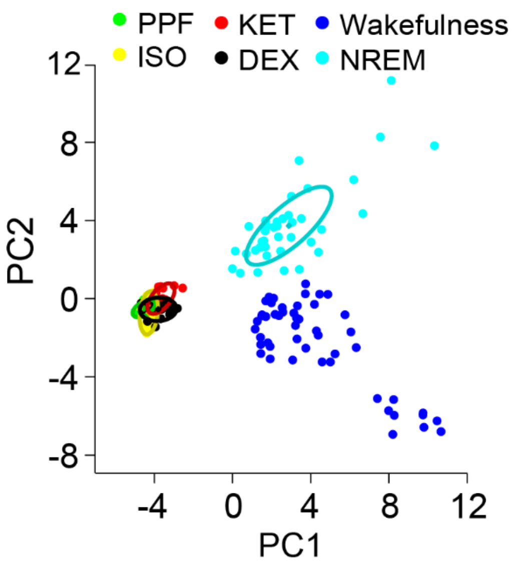

Figure S12. Anesthesia and sleep cause a distinct state of the vLPO glutamatergic neurons.

Visualization of the neural activity in the state space using PCA. Neural activity under various conditions was color-coded. The plot was constructed using data from 68 neurons that were recorded in all five conditions; each dot represents population neural activity in a 30-s bin.

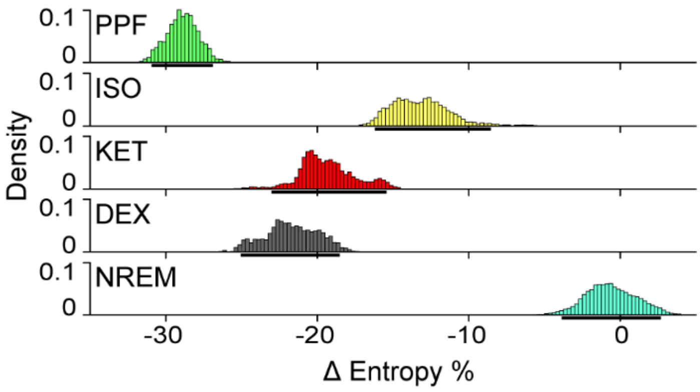

Figure S13. Anesthesia and sleep cause distinct changes in the entropy of the vLPO glutamatergic neurons.

The dataset in Figure 5B was used to calculate population entropy of the vLPO^{Glut} neurons. Δentropy (comparing with that in wakefulness): -28.9%, 95% CIs [-30.9% ~ -26.9%]; -13.2%, [-16.2% ~ -8.6%]); -19.4%, [-

23.0% ~ -15.4%]); -21.7%, [-25.1% ~ -18.5%]); and -0.61%, [-3.86% ~ 2.69%] for PPF, ISO, KET, DEX, and NREM conditions, respectively.

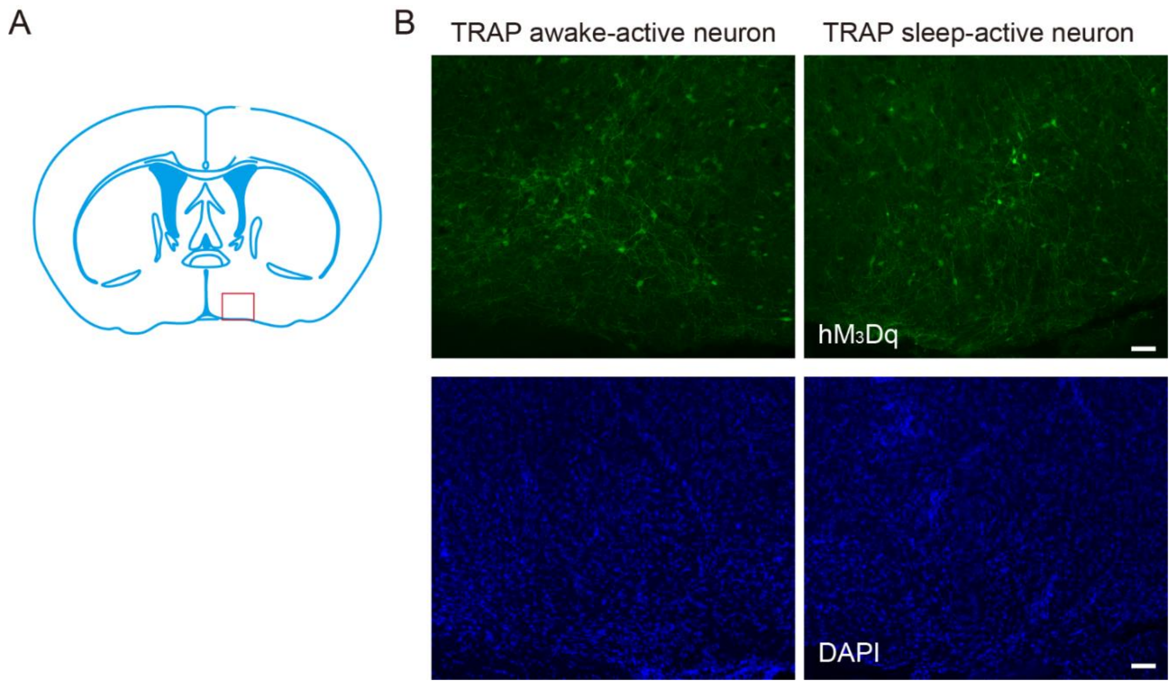

Figure S14. Histological verification of virus expression of TRAP awake-active and sleep-active neurons.

A–B) Images showing the virus expression. AAV expressing Cre-dependent DIO-hM$_3$Dq (rAAV-Ef1α-DIO-hM$_3$Dq-EYFP-WPRE-pA) was injected bilaterally injected into the target region of vLPO. Scale bars represent 50 µm.
